# Supplementary material for: Magnetically controlled multimodal motion for environmentally adaptive soft millirobots with transformable wheel-leg morphology
Source: Innovation (Camb). 2025 Oct 24;7(3):101146. doi: 10.1016/j.xinn.2025.101146 (PMC12957565; doi:10.1016/j.xinn.2025.101146)
Supplement: Document S1. Figures S1–S8, Tables S1 and S2, and Note S1 [file mmc1.pdf]

The Innovation, Volume 7

Supplemental Information

Magnetically controlled multimodal motion for environmentally adaptive soft millirobots with transformable wheel-leg morphology

Shihao Zhong, Ruhao Nie, Zhiqiang Zheng, Yaozhen Hou, Qing Shi, Qiang Huang, Toshio Fukuda, and Huaping Wang

This PDF file includes:

Note S1

Figures S1 to S8

Tables S1 to S2

Other supporting materials for this manuscript include the following:

Movies S1 to S7

SUPPLEMENTARY NOTE

S1. Fabrication process

***PDMS-LIG-xerogel composite*:** First, LIG was generated onto the surface of polyimide (PI: 63μm, Kapton, Electron® Microscopy Sciences) tape by laser scribing (wavelength, 10.6 μm; beam size, ~120 μm, Universal Laser Systems, PLS6-150D). The parameters were set as follows: laser power (4 W), laser speed (260 mm/s), dots per inch (1,000), and pulse repetition rate (10.5 kHz). The uncured PDMS (base to curing agent weight ratio of 15:1) and NdFeB hard magnetic microparticles (Magnequench GmbH, MQP-15-7, diameter 5 μm) were mixed in a 1:1 weight ratio. Then, the PDMS mixed solution was poured onto the surface of LIG, and the PDMS-LIG double-layer structure was obtained after heating and curing (80°C, 6 hours). Due to the conductive properties of the LIG layer, alginate gel was electrodeposited on its surface. Prior to electrodeposition, the LIG-PDMS composite layer was treated with plasma for 10 minutes to enhance surface activity. Next, the sodium alginate solution was evenly spread over the top of the LIG side, and then vacuumed for 10 minutes to allow the sodium alginate to penetrate the surface of the LIG. During the electrodeposition process, the LIG layer served as the bottom electrode, while ITO glass was used as the top electrode. A 4 V DC voltage was applied for 120 seconds to initiate the alginate polymerization process. After electrodeposition, the uncured solution was carefully removed by washing the composite film with distilled water three times. The alginate gel was dried at room temperature for 48 hours to form a xerogel. Note that due to the porous structure of the LIG layer, the LIG layer formed a tight cross-linked network with the immersed hydrogel and PDMS solution and resulted in a solid three-layer nested structure after curing.

***Laser Engraving-Based Xerogel Patterning*:** The 355 nm ultraviolet laser cutting system (LAJAMIN LASER) was employed to pattern the xerogel layer, enabling controlled stress distribution and programmable deformation. To selectively remove the xerogel layer, the optimized engraving parameters included a 10 A drive current, a scanning speed of 650 mm/s, and a pulse frequency of 50 kHz. Additionally, the ultraviolet laser cutting system was used to cut the required sheet shape for the three-layer composite structure. The optimized engraving parameters included a 10 A drive current, a scanning speed of 10 mm/s, and a pulse frequency of 20 kHz.

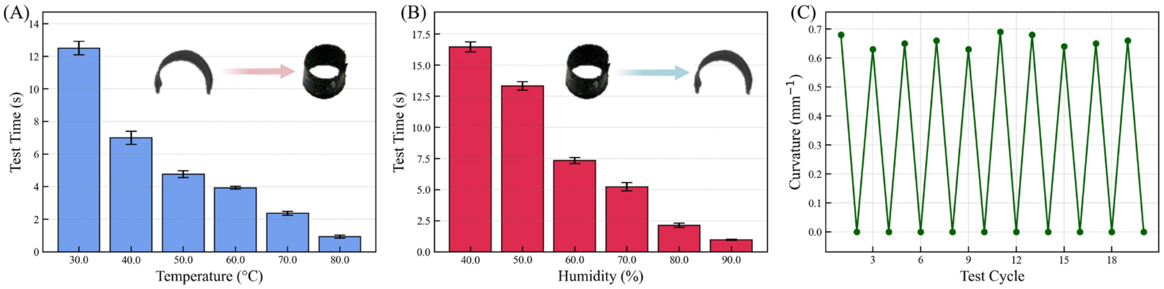

Figure S1. Response test under humidity/temperature excitation. (A) Transformation time from wheel to leg morphology under different temperature excitations. (B) Transformation time from leg to wheel morphology under different humidity excitations. (C) Results of wheel-leg shape transformation across multiple cycles in RH range 40% to 90% and temperature range 70°C to 30°C. Error bars represent SD.

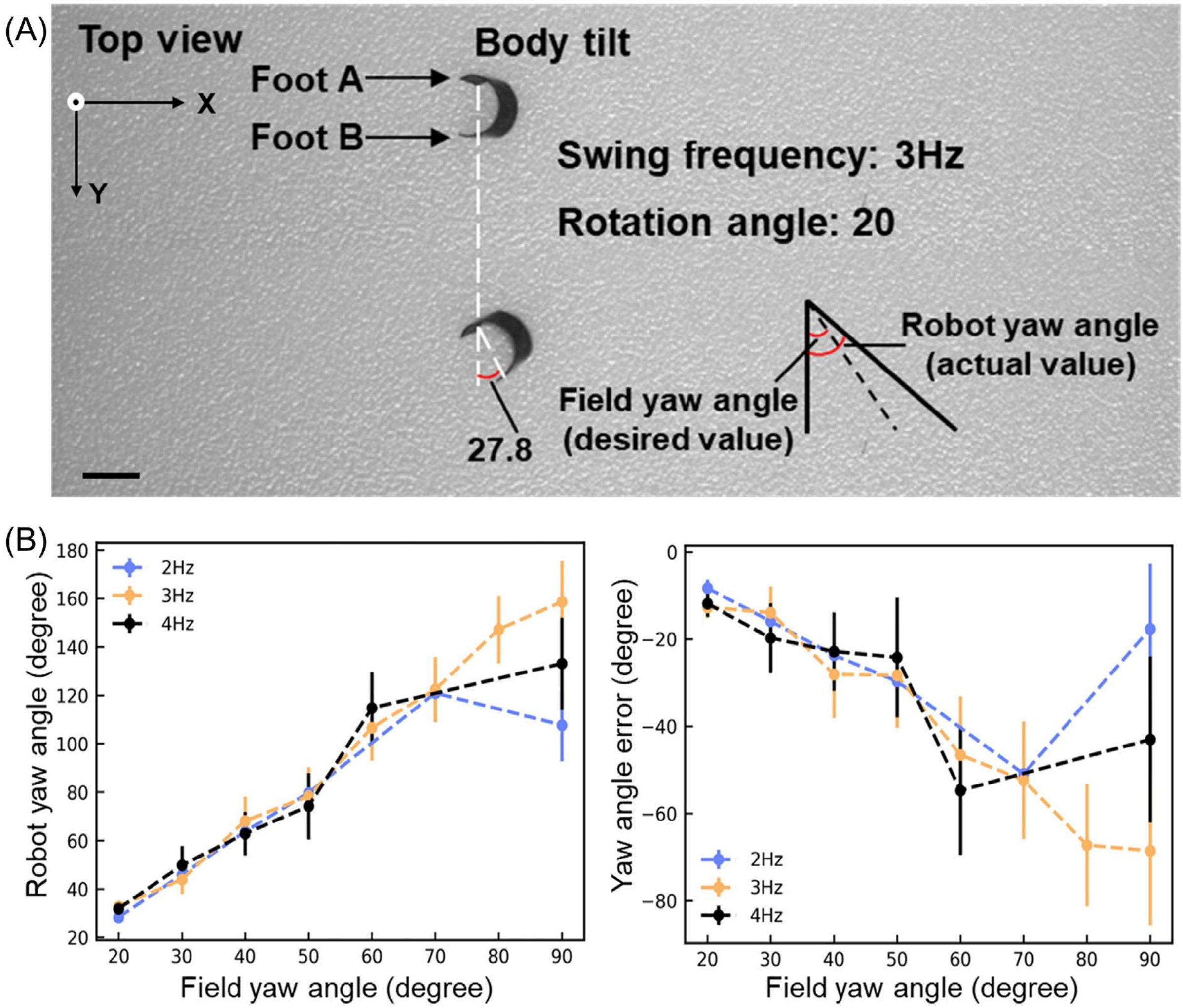

Figure S2. (A) Schematic diagram of magnetic field deflection angle and actual deflection angle of the millirobot. Scale bar: 3 mm. (B) Test results of the relationship between the magnetic field deflection angle and the actual deflection angle of the millirobot. Error bars represent SD.

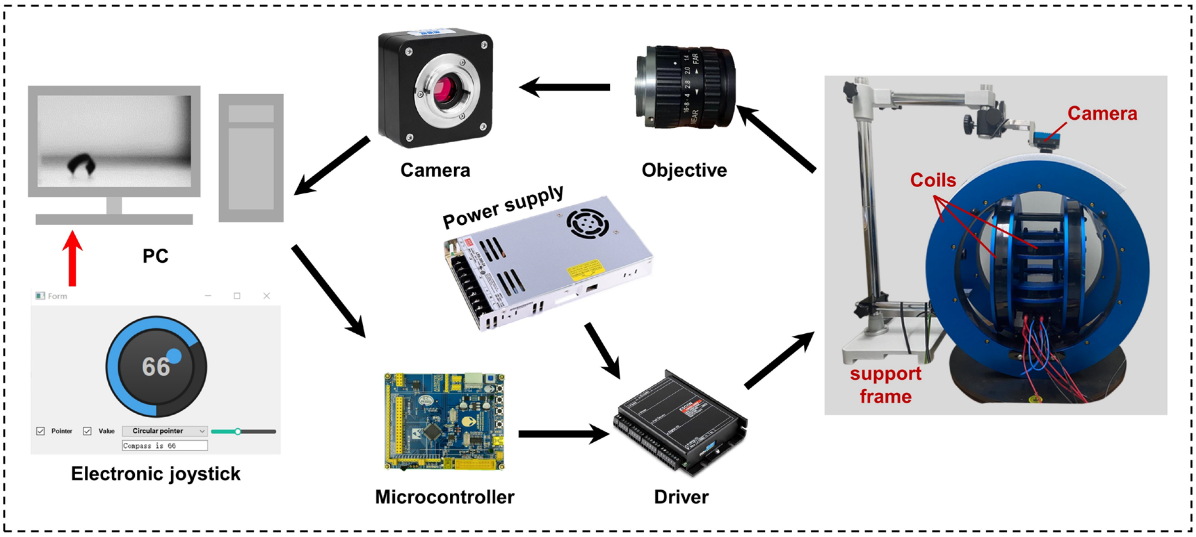

Figure S3. Experimental setup system.

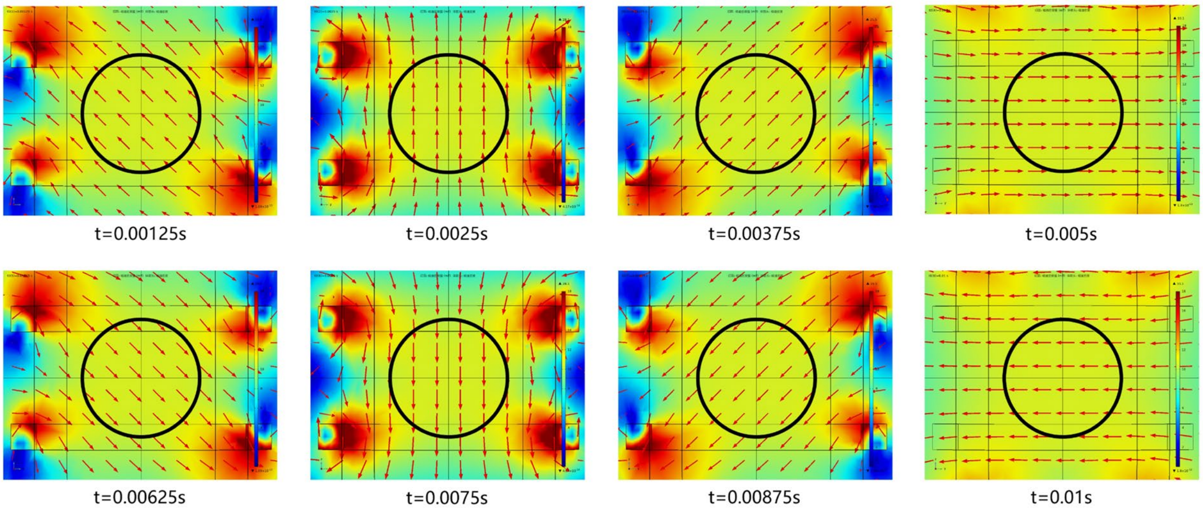

Figure S4. Simulation results of rotating magnetic field.

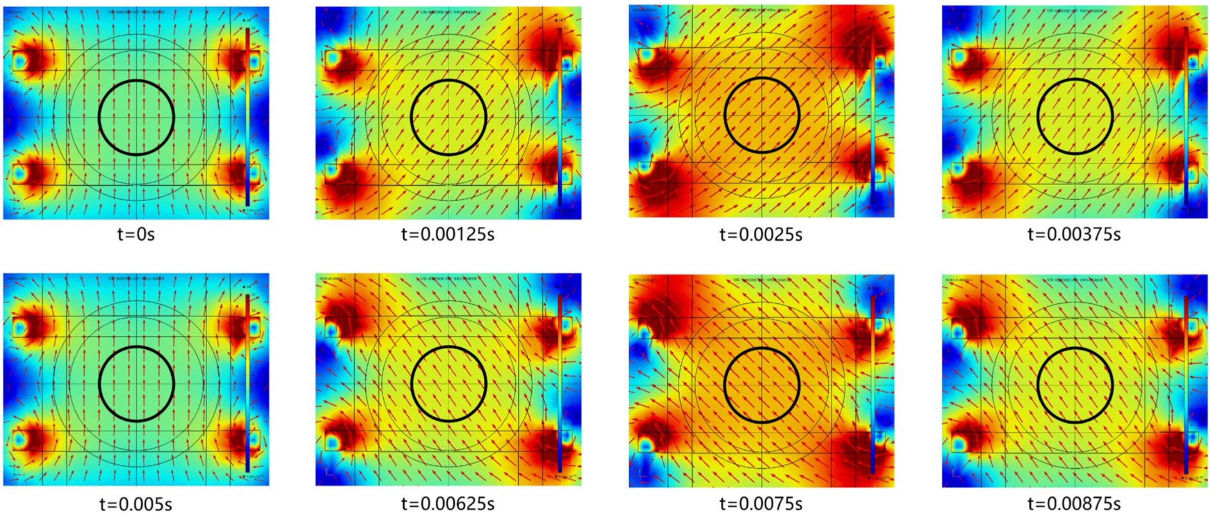

Figure S5. Simulation results of swing magnetic field.

Table S1. Electromagnetic coil parameters

Parameters	X	Y	Z
Number of turns	281	398	262
Equivalent radius (mm)	236	167	110
Wire diameter (mm)	Φ1.95	Φ1.4	Φ1.4
Coil cross section (mm*mm)	35*35	30*30	24*24
Maximum continuous current (A)	10	5	5
Magnetic flux density (mT)	107	107	107
Cold resistance (Ω)	4.8	9.3	4
Inductance (mH)	166	221	58.3

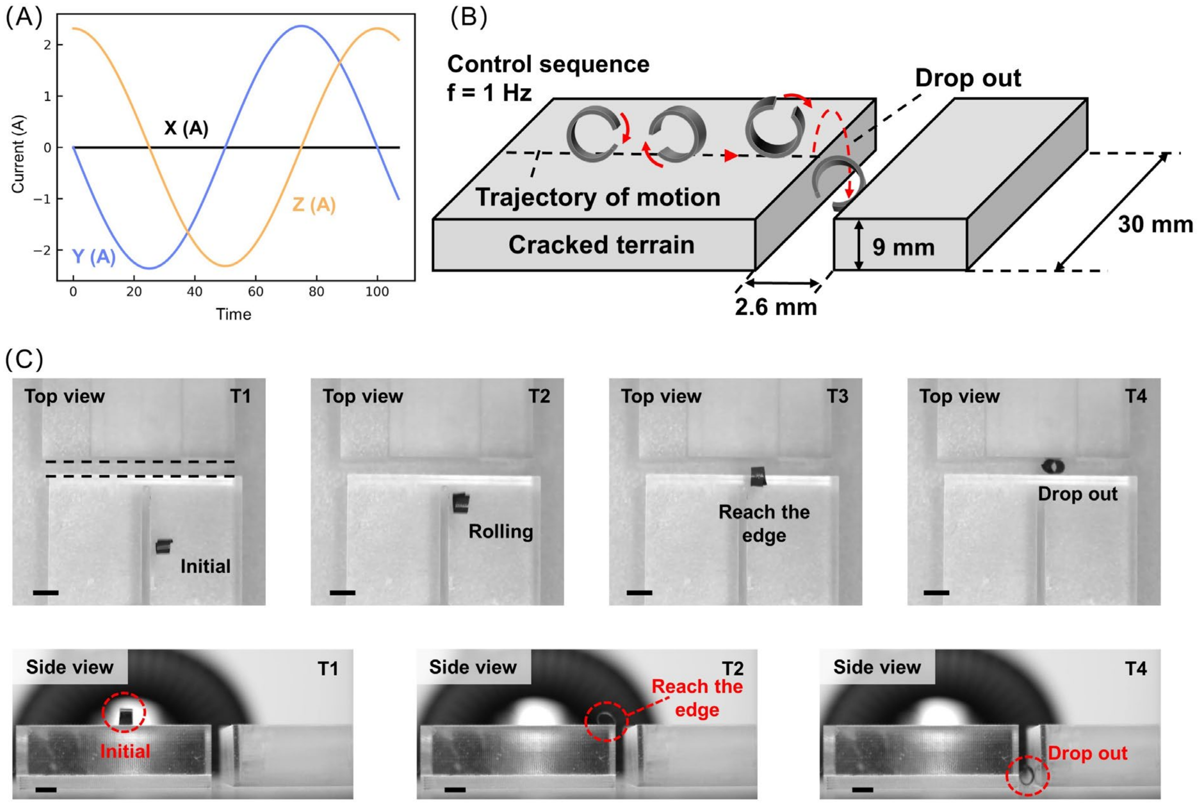

Figure S6. Wheeled millirobots traversing the gap. (A) The currents control signal of generating rotating magnetic field for rolling. (B) Diagram of the millirobot crossing the slit and the schematic of the geometries and sizes in this terrain model. The rolling frequency of the magnetic field is 1 Hz. (C) Millirobot motion state sequence diagram at different time. Scale bar: 5 mm.

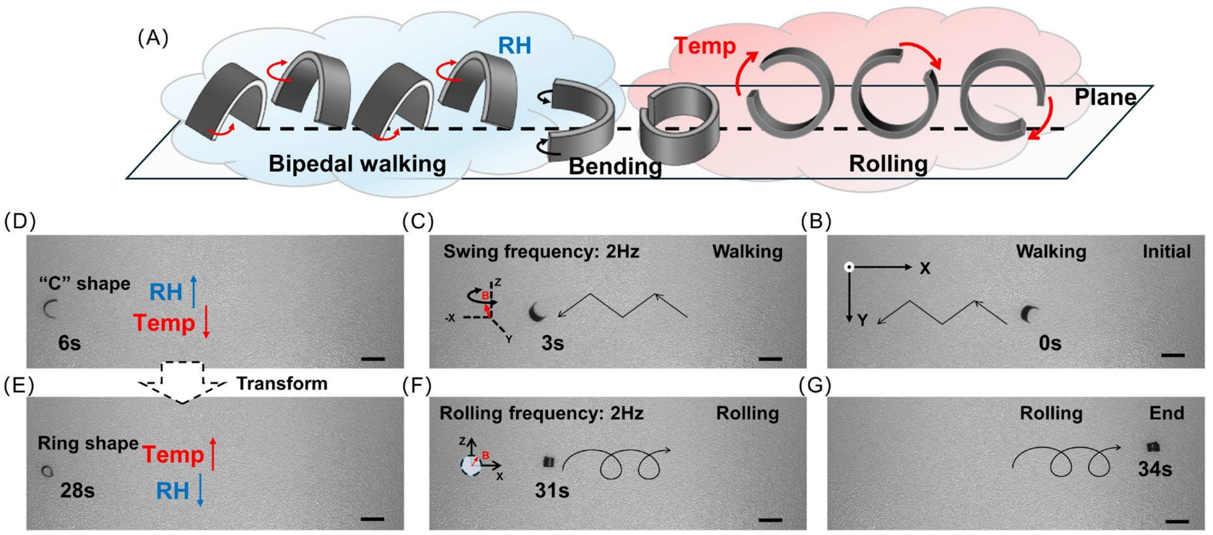

Figure S7. Millirobot dual modality transformation. (A) Schematic representation of the millirobot morphological transformation. (B)-(C) Snapshots of the legged millirobot walking. (D)-(E) Legged millirobot is converted to wheeled millirobot. (F)-(G) Snapshots of the wheeled millirobot rolling. Scale bar: 5 mm.

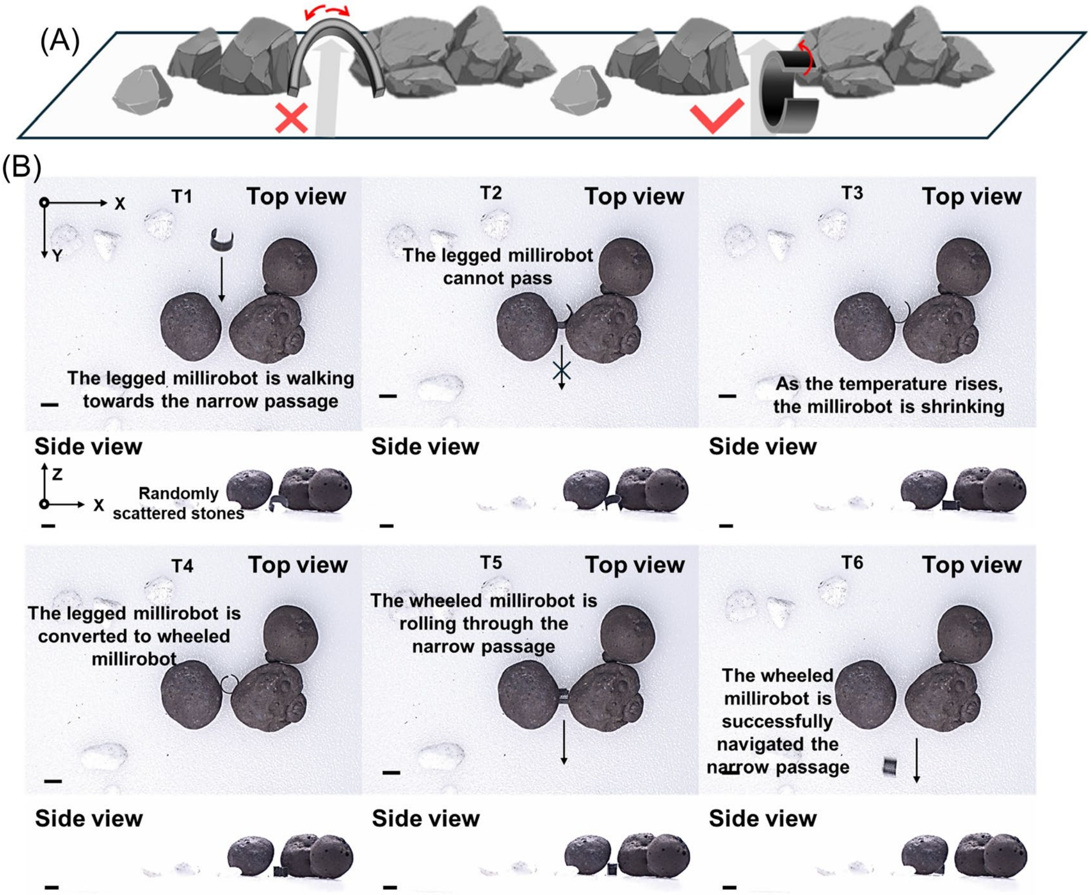

Figure S8. The millirobot passed through unstructured narrow slits based on morphological transformation. (A) Schematic of the mobility of different morphologies of the millirobot. (B) Snapshots of the wheel-legged millirobot passed through unstructured narrow channels. Scale bar: 3 mm. The average radius of the stone is 9 mm.

Table S2. Robot performance comparison table

	**Max. Speed**	**Complex Terrain Adaptability**	**Controllability**	**Payload/Function**
Wang et al.[1]	Approximately 1.16 Body Lengths/s	Vertical plane, Narrow entries/gaps	Preset signal/manual control	2.5 times its self-weight
Huang et al.[2]	Approximately 1.83 Body Lengths/s	Sand, Muddy, Slope, Gap	Preset signal/manual control	5 times its self-weight
Wang et al.[3]	Approximately 100.6 Body Lengths/s	Jump over the railings of different heights	Preset signal/manual control	Underwater microrobot recovery
Mao et al.,[4]	Approximately 70 Body Lengths/s	Jump over obstacles that are up to 2/3 of your own height	Preset signal/manual control	Cargo transport
Xin et al.[5]	Approximately 0.67 Body Lengths/s	Slit, Stair	Linear closed loop	Drug/cell transport
Our work	Approximately 56 Body Lengths/s	Slope, Curved surface, Stair, Slit, Gap	Nonlinear closed-loop control	13 times its self-weight

References

1. Wang X., Li S., Chang J.C., et al. (2024). Multimodal locomotion ultra-thin soft robots for exploration of narrow spaces. *Nat. Commun.* 15, 6296.
2. Huang H., Feng Y., Yang X. et al. (2022). An insect-inspired terrains-adaptive soft millirobot with multimodal locomotion and transportation capability. *Micromachines* 13, 1578.
3. Mao G., Schiller D., Danninger D., Hailegnaw B., et al. et al. (2022). Ultrafast small-scale soft electromagnetic robots. *Nat. Commun.* 13, 4456.
4. Wang, X., Xia N., Pan C. et al. (2025). Beyond surface tension-dominated water surface jumping. *Nat. Commun.* 16, 3034.
5. Xin Z., Zhong S., Wu A. et al. (2025). Dynamic control of multimodal motion for bistable soft millirobots in complex environments. *IEEE Trans.* Robot. 41, 2662–2676.
