# Supplementary material for: Magnetically controlled multimodal motion for environmentally adaptive soft millirobots with transformable wheel-leg morphology
Source: Innovation (Camb). 2025 Oct 24;7(3):101146. doi: 10.1016/j.xinn.2025.101146 (PMC12957565; doi:10.1016/j.xinn.2025.101146)
Supplement: Document S2. Article plus supplemental information [file mmc9.pdf]

ARTICLE Automation

OPEN ACCESS

www.the-innovation.org

Magnetically controlled multimodal motion for environmentally adaptive soft millirobots with transformable wheel-leg morphology

Shihao Zhong,[1,2] Ruhao Nie,[1,2] Zhiqiang Zheng,[3,*] Yaozhen Hou,[1,2] Qing Shi,[1,2] Qiang Huang,[1,2] Toshio Fukuda,[4] and Huaping Wang[1,2,*]

*Correspondence: zhzheng@cityu.edu.hk (Z.Z.); wanghuaping@bit.edu.cn (H.W.)

Received: May 7, 2025; Accepted: October 17, 2025; Published Online: October 24, 2025; https://doi.org/10.1016/j.xinn.2025.101146

© 2025 The Author(s). Published by Elsevier Inc. on behalf of Youth Innovation Co., Ltd. This is an open access article under the CC BY license (http://creativecommons.org/licenses/by/4.0/).

GRAPHICAL ABSTRACT

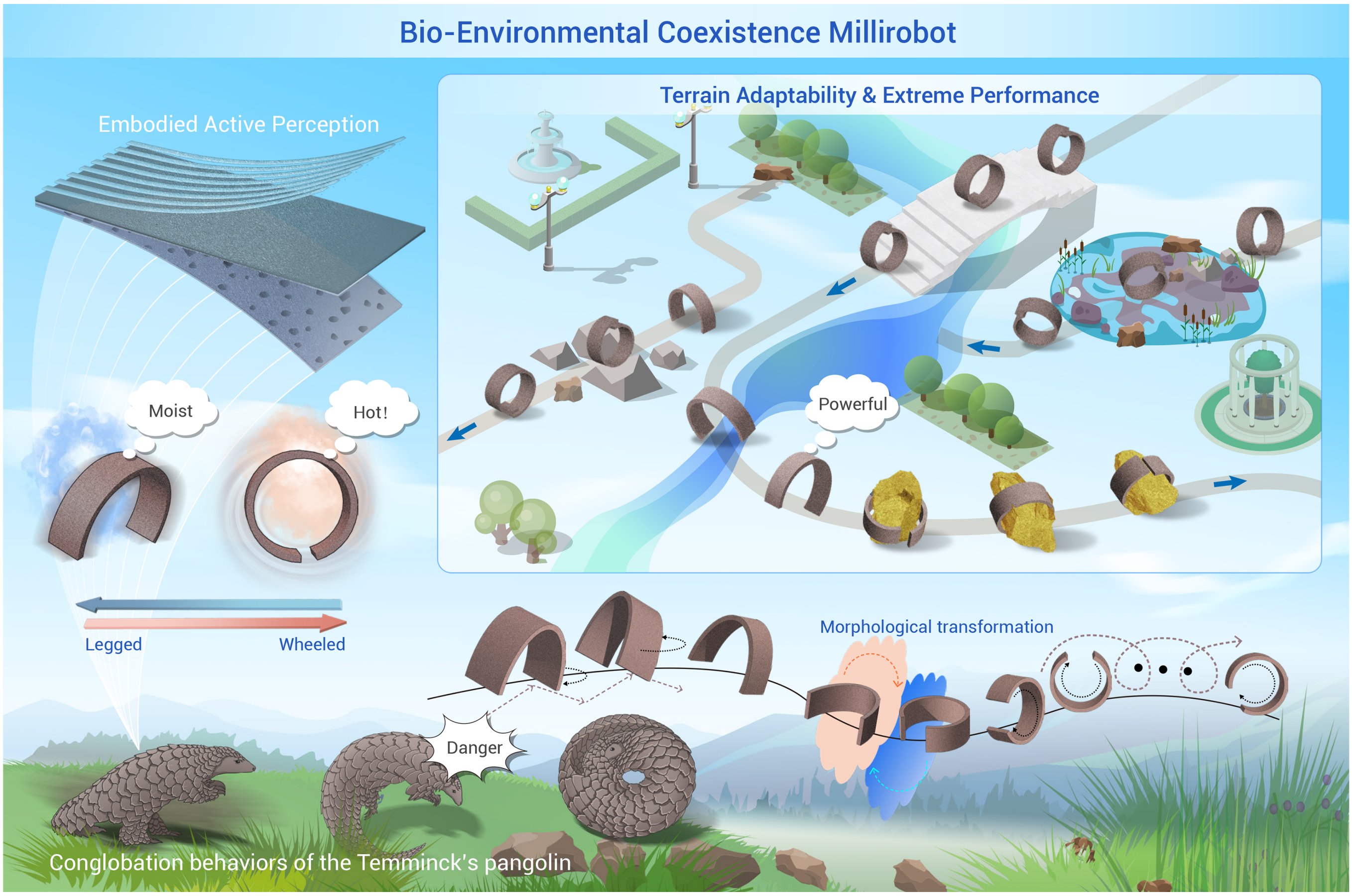

PUBLIC SUMMARY

- Inspired by animal conglobation, adaptive millirobots with transformable wheel-leg morphology are designed.
- It senses ambient humiture to adapt and hold its shape, moving in multiple modes via magnetic fields.
- It is capable of autonomous bipedal walking and wheeled rolling across unstructured terrains.
- It can transport cargo far heavier than itself, for future micro-object manipulation in confined spaces.

OPEN ACCESS

Automation ARTICLE

Magnetically controlled multimodal motion for environmentally adaptive soft millirobots with transformable wheel-leg morphology

Shihao Zhong,[1,2] Ruhao Nie,[1,2] Zhiqiang Zheng,[3,*] Yaozhen Hou,[1,2] Qing Shi,[1,2] Qiang Huang,[1,2] Toshio Fukuda,[4] and Huaping Wang[1,2,*]

[1]Intelligent Robotics Institute, School of Mechatronical Engineering, Beijing Institute of Technology, Beijing 100081, China

[2]Key Laboratory of Biomimetic Robots and Systems, Beijing Institute of Technology, Ministry of Education, Beijing 100081, China

[3]Department of Biomedical Engineering, City University of Hong Kong, Hong Kong SAR 999077, China

[4]Department of Micro-Nano Systems Engineering, Nagoya University, Furo-cho, Chikusa-ku, Nagoya, Aichi 464-8603, Japan

*Correspondence: zhzheng@cityu.edu.hk (Z.Z.); wanghuaping@bit.edu.cn (H.W.)

Received: May 7, 2025; Accepted: October 17, 2025; Published Online: October 24, 2025; https://doi.org/10.1016/j.xinn.2025.101146

© 2025 The Author(s). Published by Elsevier Inc. on behalf of Youth Innovation Co., Ltd. This is an open access article under the CC BY license (http://creativecommons.org/licenses/by/4.0/).

Citation: Zhong S., Nie R., Zheng Z., et al., (2026). Magnetically controlled multimodal motion for environmentally adaptive soft millirobots with transformable wheel-leg morphology. The Innovation **7(3)**, 101146.

Small-scale soft robots with high morphological flexibility show significant potential for precise operation and sensing in confined environments. However, due to the coupled driving mechanism and the influence of environmental disturbances, the highly adaptable and stable navigation across diverse terrains through multimodal motion, which involves morphing shape and maintaining the reshaped configuration, still presents a major challenge for soft millirobots. Here, we develop a multi-stimuli-responsive millirobot with a multimodal locomotion adaptive control method, enhancing environmentally synergistic interactions and tasking capabilities. Constructed from materials responsive to temperature, humidity, and magnetic fields, the millirobot precisely navigates unstructured environments and independently controls deformation and locomotion. Theoretical models guide its polymorphic locomotion with optimal actuating parameters, such as bipedal walking in the two-leg mode and rolling in the wheel mode. A hierarchical dual-layer path-following controller manages path information and adjusts movement patterns. Experiments demonstrate the millirobot's environmental adaptability, morphological complementarity, and functional diversity. With various locomotion modes across different morphologies, the millirobot can traverse slopes, curved surfaces, stairs, slits, and gaps. It also performs tasks, such as cargo capture and transport, through morphological transformation. The proposed multimodal motion strategy based on polymorphism makes the soft millirobot a promising candidate for applications in micro-object manipulation and crevice inspection at confined, varied, and unstructured terrains.

INTRODUCTION

Due to the remote drive capabilities and non-invasive access in closed and narrow environments, untethered millirobots are gaining increasing interest in sensing, control, and micromanipulation applications.[1–8] In comparison to rigid structures, soft millirobots offer enhanced safety and adaptability in interactions with diverse environments.[1–6] Moreover, the multiple degrees of freedom inherent in deformable soft millirobots significantly enhance their ability to navigate confined spaces, making them a key focus in milli-scale robotics research.[9–11] Flexible navigation through unstructured and complex terrains demands that soft millirobots have enhanced motion efficiency and environmental adaptability. Researchers employ elaborate structural designs for soft millirobots to enable task-specific movement modes adaptable to various environmental conditions, such as wheel rolling,[12] legged walking,[13,14] spiral rotation,[15,16] and elastomer jumping.[17] However, millirobots with a single mode of motion have limited mobility because they have trouble negotiating varied obstacles and adapting to changes in texture or material within unstructured environments. Therefore, developing multimodal motion capabilities holds significant potential for improving the performance of soft millirobots.

Driven by constant periodic actuation signals from the specific driving source, soft millirobots maintain their dynamic deformation, resulting in diverse motion modes such as undulation, creep, walking, rolling, and swimming. Multimodal motion in soft millirobots featuring a custom-magnetized curve is achieved through the application of dynamic magnetic fields, which are generated by adjusting either the input current pattern of the electromagnetic coil system[11,18,19] or the motion trajectory of the permanent magnet in three-dimensional (3D) space.[20–22] The temperature/humidity-responsive soft millirobot,[23–25] inspired by the water-absorbing expansion and dehydration contraction behavior of fiber plants, reciprocally extends, contracts, bends, or distorts its body to perform multimodal actions. In addition, the local or anisotropic contraction and expansion behaviors of soft materials based on chemistry,[26,27] sound,[28,29] and light[30,31] also offer effective methods for enabling multimodal motion in soft millirobots. However, the multimodal locomotion of soft millirobots with only a single stable geometric configuration relies on specific external control signals continuously input to simultaneously sustain structural deformation and coordinate movement through coupled actuation mechanisms. When disturbances arise from the drive system—such as magnetic field inhomogeneity and hysteresis—as well as from the external environment—such as terrain structure and changes in friction—control input fluctuations or non-coordinated interactions between the soft millirobot and the environment may occur, potentially inducing transient instabilities manifesting as abrupt deformations, locomotion intermittency, or sustained oscillations. Therefore, the decoupling of morphological reconstruction and motion control emerges as a critical strategy for achieving stable, adaptive, and efficient multimodal locomotion in soft millirobots.

The capacity of organisms with high morphological flexibility to actively adjust and maintain their body structure and behavior in response to changing environments and external disturbances serves as inspiration. Pangolins,[32] Armadillidiidae,[33] armadillo,[34] hedgehogs,[35] etc., can achieve stable walking on unstructured terrain and can curl their bodies into a ringlike shape (i.e., conglobation behaviors[36]) for self-protection, hunting, and escape. Researchers have attempted to mimic these mechanisms of morphological transformation to enable steady-state structural reconfiguration in soft millirobots, resulting in more stable multimodal motion. One of the most popular research approaches involves developing materials with multiple excitation response properties,[37–39] allowing for morphological control through the introduction of additional driving sources. Numerous soft materials, employing dual- or multi-excitation coupling mechanisms,[40–45] such as magnetic, thermal, hydro, electrical, optical, chemical, and others, have been reported in abundance, demonstrating remarkable actuation and deformation capabilities. Through an iterative trial-and-error process, excitation signal sequences are adjusted to optimize movement across different body morphologies and motion modes. As the number of morphologies and locomotion modes increases, manually tuned actuation parameters and timing preset driver parameters exhibit inefficiency, lack of flexibility, and poor control accuracy, making performing specific tasks, such as grabbing and releasing or accurate delivery, challenging.[46,47] Therefore, developing an effective multimodal dynamic motion model and achieving accurate motion control for polymorphic millirobots remain significant challenges.

Here, we design, fabricate, and model a multi-stimuli-responsive wheel-legged millirobot, enabling high flexibility and adaptability in confined environments through polymorphic transformation and multimodal locomotion. The sheet-shaped millirobot is constructed by stacking and patterning three layers (two actuating layers and a connection layer) of functional materials in two dimensions, with parted actuating layers contributing to independent motion control via a magnetic field and deformation control via temperature/humidity excitation. The motion pattern, inspired by the conglobation behaviors of the Temminck's pangolin,[48] is designed to accommodate both bipedal walking in a leg morphology and rolling in a wheel morphology (Figure 1). Theoretical models of the actuation mechanisms and locomotion behaviors of the polymorphic millirobot were developed to establish optimal actuating parameters,

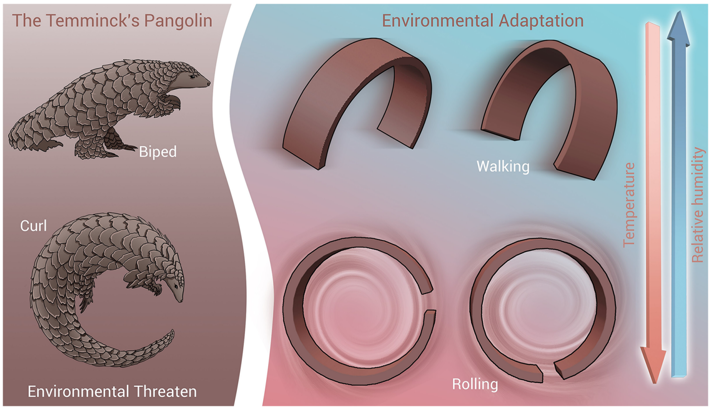

Figure 1. Bio-conglobation behaviors Polymorphic millirobots with multimodal movement capabilities, inspired by the Temminck's pangolin.

thereby facilitating precise motion control and enabling a variety of functions. For high-precision path-following maneuverability, a hierarchical dual-layer path-following controller is designed, comprising a switching controller that governs millirobot morphology and the driving magnetic field and a cascade controller that autonomously regulates the orientation of the magnetic field within a 3D space. Experiments demonstrate that the polymorphic millirobot can climb slopes and cross gaps using a leg morphology, traverse curved terrain and climb stairs using a wheel morphology, and perform cargo capture and transport through morphological transformation and multimodal locomotion, showcasing its high environmental adaptability and enhanced tasking capabilities. We anticipate that soft millirobots, which achieve environmentally adaptive behavior through their transformable morphology and an automatic multimodal motion control strategy, can be deployed in various confined and unstructured environments for micro-object pick-and-place and precision equipment maintenance tasks in the future.

MATERIALS AND METHODS

Design and fabrication

The morphologically convertible millirobot was fabricated as a planar, three-layer laminate comprising a magnetic elastomer, laser-induced graphene (LIG), and an alginate xerogel (Figure 2A). The magnetic elastomer, a composite of polydimethylsiloxane (PDMS) and magnetic microparticles (MMPs), was directionally magnetized to enable motion control. The porous LIG layer served as an intermediate scaffold, forming a robust cross-linked network with both the hydrogel and the uncured magnetoelastomer to create a solid, nested structure upon curing.

To fabricate the structure, the uncured PDMS mixture was first poured onto the LIG surface and heat cured, forming a magnetic elastomer-LIG bilayer. Subsequently, leveraging the conductivity of the LIG, an alginate gel was electrodeposited onto the opposing surface. This alginate hydrogel was then dried at room temperature to form the final xerogel layer. The conversion from hydrogel to xerogel introduced a significant prestress between the xerogel and the elastomer layers. This stored stress could be released through humidification-drying cycles, inducing a 2D-to-3D structural transformation. Thus, the alginate xerogel layer provides fast and reversible deformation control in response to temperature and humidity. Detailed fabrication parameters are available in Note S1.

Due to non-uniform internal stress, the as-fabricated xerogel film initially exhibited asymmetric deformation. To achieve programmable deformation, laser patterning was used to selectively ablate the xerogel layer, thereby tailoring the stress distribution and the resulting curvature. Specifically, to achieve a symmetric, circular contraction, the xerogel was patterned into a series of parallel strips, creating a "zebra crossing" pattern that homogenized the internal stress along a single axis. Detailed laser patterning parameters are provided in Note S1. The actuation mechanism is as follows: in a high-humidity (or low-temperature) environment, the xerogel strips absorb water and expand, causing the structure to become planar. Conversely, in a low-humidity (or high-temperature) environment, water evaporation causes the xerogel to shrink, inducing a bending moment. Throughout this hygroscopic expansion and contraction, each rectangular xerogel strip undergoes an anisotropic dimensional change, with a greater change in length than in width. This anisotropy ensures that the bending direction is predictable and consistently occurs along the longitudinal axis of the strips.

Deformation mechanical analysis

Deformation geometry relation: the moduli of PDMS and xerogel are significantly different, with high-modulus dry gel providing the driving force and low-modulus PDMS allowing large deformations. Due to the xerogel being uniformly removed, the active layer is homogenized as sketched in Figure 2B, and the effective active thickness h_2 is

$$h_2 = \tilde{h}_2 \frac{w_{xerogel}}{w_{xerogel}+w_{empty}}. \quad \text{(Equation 1)}$$

We assume that the millirobot's thickness and cross-section remain unchanged during shape deformation. The geometric relation of the deformation is as follows:

$$R\theta = L + \Delta L, \text{ and} \quad \text{(Equation 2)}$$

$$[R + (h_1 + h_2)]\theta = L, \quad \text{(Equation 3)}$$

where R is the radius of the curvature; θ is the bending angle; L is the length of the original sample beam; ΔL is the change in length of the active layer, where elongation is positive and shortening is negative; and h_x is the thickness of constitutive layer $x = 1, 2$.

From Equations 2 and 3,

$$\theta = \frac{-\Delta L}{h_1+h_2}, \text{ and} \quad \text{(Equation 4)}$$

$$k = \frac{-\Delta L}{(h_1+h_2)(L+\Delta L)}. \quad \text{(Equation 5)}$$

Humidity-curvature relation: by using the diffusion transport of water in the hygroscopic active layer, the transient bending response of the double-layer actuator can be calculated using the elastic theory[23] (Figure 2B). The 1D diffusion of water molecules follows

$$\partial\phi/\partial t = D\partial^2\phi/\partial z^2, \quad \text{(Equation 6)}$$

where $\phi(z,t)$ is the water concentration, t denotes time, and z is the thickness.

If the initial concentration is $\phi(z,0) = \phi_0$, the outer surface concentration is $\phi(0,t) = \phi_\infty$, and the boundary condition of the interface between the active layer and the inactive layer is $\partial\phi(h,t)/\partial z = 0$, the concentration distribution in the active layer ($0 < z < h$) is

$$\phi(z,t) = \sum_{n=0}^{\infty}\left[\frac{-2(\phi_0-\phi_\infty)}{\left(n+\frac{1}{2}\right)\pi} e^{-\lambda_n^2 t} \sin\frac{\left(n+\frac{1}{2}\right)\pi z}{h}\right] + \phi_\infty, \quad \text{(Equation 7)}$$

where $\lambda_n = D^{1/2}(n + 1/2)\pi/h$ and ϕ_∞ is the environmental humidity.

$$\varepsilon(z,t) = \varepsilon_0 - \kappa z - \varepsilon_h, \text{ and} \quad \text{(Equation 8)}$$

$$\text{s.t. } \varepsilon_h = \alpha\phi,$$

where $\varepsilon(z,t)$ is the total strain in the active layer, ε_0 is the reference strain at the reference plane ($z = 0$), z is the distance from the reference plane, κ is the bending curvature, ε_h is the local moisture concentration that induces the hygroscopic strain, and α is the hygroscopic expansion coefficient.

In the absence of any external load, the force F and the moment M can be written as

$$F = \int \sigma dz = 0, \text{ and} \quad \text{(Equation 9)}$$

$$M = \int \sigma z dz = 0, \quad \text{(Equation 10)}$$

where the local stress $\sigma = E\varepsilon$, where E is Young's modulus.

The planar millirobot was designed with a length of L = 8 mm and a width of w = 2 mm to exhibit distinct, humidity-dependent morphologies. In high humidity (relative humidity [RH] = 90%), it maintains a flat, strip-like shape, while in low humidity (RH = 20%), it fully contracts into a ring. At ambient room conditions (RH = 40%–60%), it spontaneously forms a stable C-shape, which serves as its default legged configuration (Figures 2C and 2D). This programmable deformation was achieved by engineering the layered structure. Based on theoretical calculations, the inactive layer thickness was set to approximately 210 μm. The 280 μm active xerogel layer was laser patterned with a zebra crossing motif (width ratio ≈ 0.5), which reduced its effective thickness to approximately 140 μm. This design, validated by both theoretical and experimental results (Figure 2D), produced the desired deformation characteristics with significantly improved control and

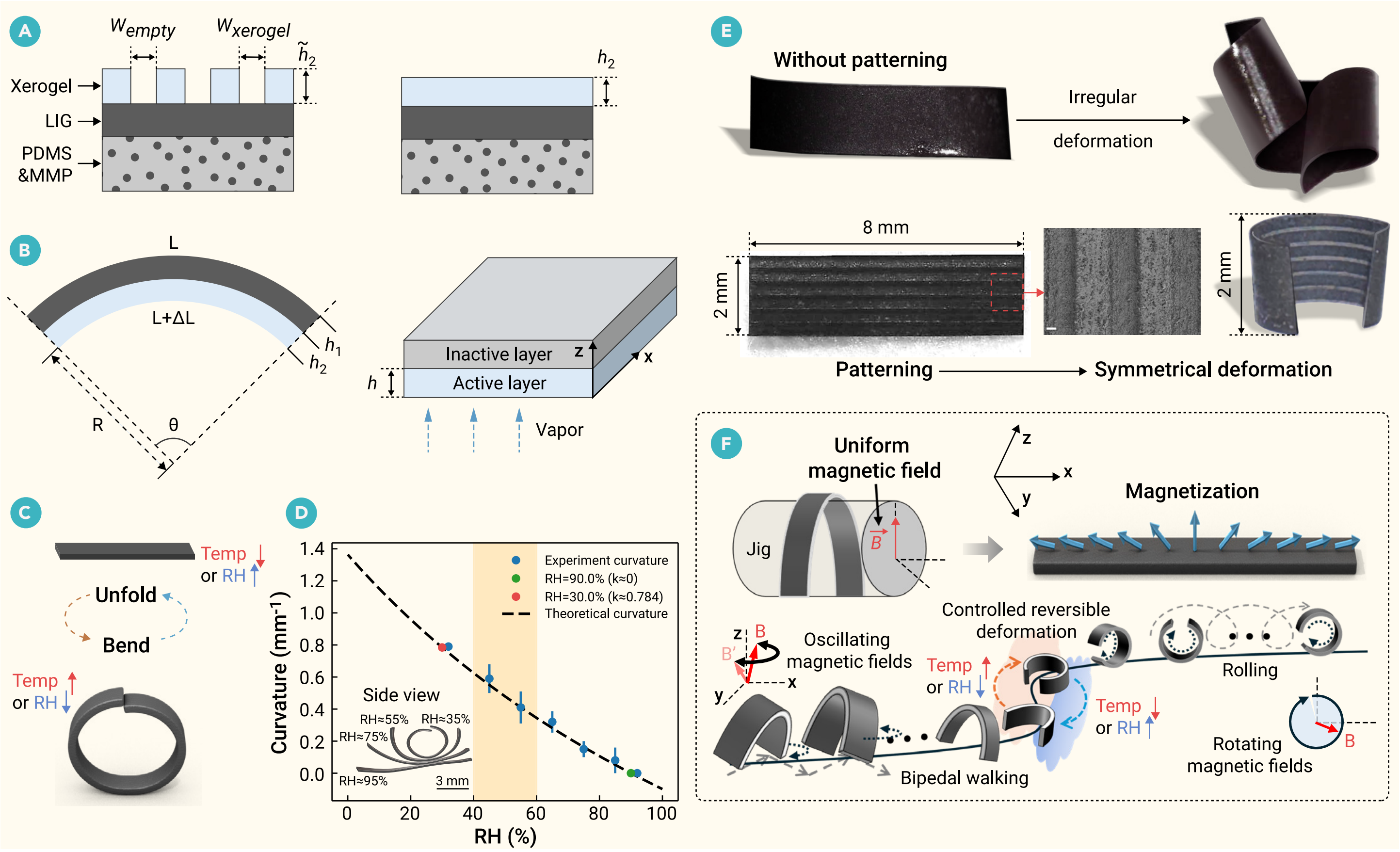

Figure 2. Design and actuation principle of the stimuli-responsive millirobot (A) The millirobot is designed from a trilayer structure (left), which is modeled to define its effective active thickness (right). (B) The bending geometry and its corresponding coordinate system. (C and D) Deformation is stimulated by changes in temperature and humidity (C), and the quantitative relationship between environmental humidity and robot curvature is plotted in (D). (E) Laser patterning is critical for controlled, symmetrical deformation, as shown by the comparison between unpatterned and patterned structures. (F) The overall motion control strategy, based on a predefined magnetization profile. Error bars in (D) represent SD. Scale bar, 30 μm.

regularity compared to unpatterned versions (Figure 2E). To enable locomotion, the C-shaped millirobot was placed in a non-magnetic cylindrical mold and subjected to an 800 mT directional magnetic field. The field was applied perpendicular to the C-shape's opening, imprinting a sinusoidal magnetization profile along the robot's length (Figure 2F).

This overall design is crucial for two reasons. First, it decouples control, allowing environmental stimuli (temperature/humidity) to govern shape morphing while an external magnetic field independently controls rigid body motion. Second, this single magnetization pattern enables multimodal locomotion: the legged C-shape performs a bipedal walk, while the wheeled ring shape achieves fast rolling. The dynamics of this wheel-leg transformation were found to be stable and reversible over multiple cycles, with transformation rates increasing at higher temperatures and humidity levels (Figures S1A and S1B). The contraction phase was observed to be faster than the expansion phase. Unless otherwise specified, the legged morphology referred to in this paper is the stable C-shape formed at room temperature.

Movement analysis and characterization

Bipedal walking gait of the legged millirobot. The legged millirobot walks by swinging its two legs in response to a swing magnetic field $\boldsymbol{B_o}$. Bipedal walking includes four basic states: stand upright, body lean, move left, and move right (Figure 3A). First, a constant uniform magnetic field orients the millirobot to an upright, forward-leaning position. As the magnetic field swings, the millirobot alternately lifts its left and right feet to walk forward. The forward lean intentionally uses gravity to break the rotational symmetry, enabling one leg to swing forward while the other acts as a support.

Mathematically, the sequence of $\boldsymbol{B_o}$ described above can be represented as

$$\boldsymbol{B_o}(t) = \begin{pmatrix} B_x \\ B_y \\ B_z \end{pmatrix} = B \begin{pmatrix} |\cos(2\pi ft)| \\ -\sin(2\pi ft) \\ \kappa \end{pmatrix}, \qquad \text{(Equation 11)}$$

where $\omega = 2\pi ft$ is the rotation angle. f and κ are used to adjust the oscillation frequency and the angle of the magnetic field, respectively.

Based on the balance of magnetic torque and gravitational moment, the relationship between the pitch angle α_r of the millirobot and the pitch angle α_B of the external magnetic field in the body lean state is obtained:

$$\alpha_B = \tan^{-1}\frac{\sin\alpha_r - \frac{GL}{2MB}}{\cos\alpha_r}, \quad and \qquad \text{(Equation 12)}$$

$$s.t.\ \alpha_r > \sin^{-1}\frac{GL}{2MB},$$

where M is the magnetic moment of the millirobot, G is the gravity acting on the millirobot, and L is the body length of the millirobot.

The swinging process of the millirobot can be simplified as two legs swinging alternately, with the single-leg swing approximated as an inverted pendulum model with the center of gravity at the top of the swinging leg (Figure 3B). The kinetic model is as follows:

$$\ddot{\boldsymbol{x}}_{\boldsymbol{m}} = \frac{\boldsymbol{F_x}}{m}, \quad and \qquad \text{(Equation 13)}$$

$$\ddot{\boldsymbol{z}}_{\boldsymbol{m}} = \frac{\boldsymbol{F_z}}{m} - \boldsymbol{g}, \qquad \text{(Equation 14)}$$

where $\boldsymbol{F_{xz}}$ is the component of magnetic force on the x and z axes, m is the mass of the millirobot, $\boldsymbol{g}$ is the acceleration due to gravity, and $\ddot{\boldsymbol{x}}_{\boldsymbol{m}}$ and $\ddot{\boldsymbol{z}}_{\boldsymbol{m}}$ are the accelerations of the millirobot on the x and z axes, respectively.

The leg length is calculated based on the centroid position (x_m,z_m) and the landing position (x_F,z_F):

$$L = \sqrt{(x_F - x_m)^2 + (z_F - z_m)^2}. \qquad \text{(Equation 15)}$$

www.the-innovation.org

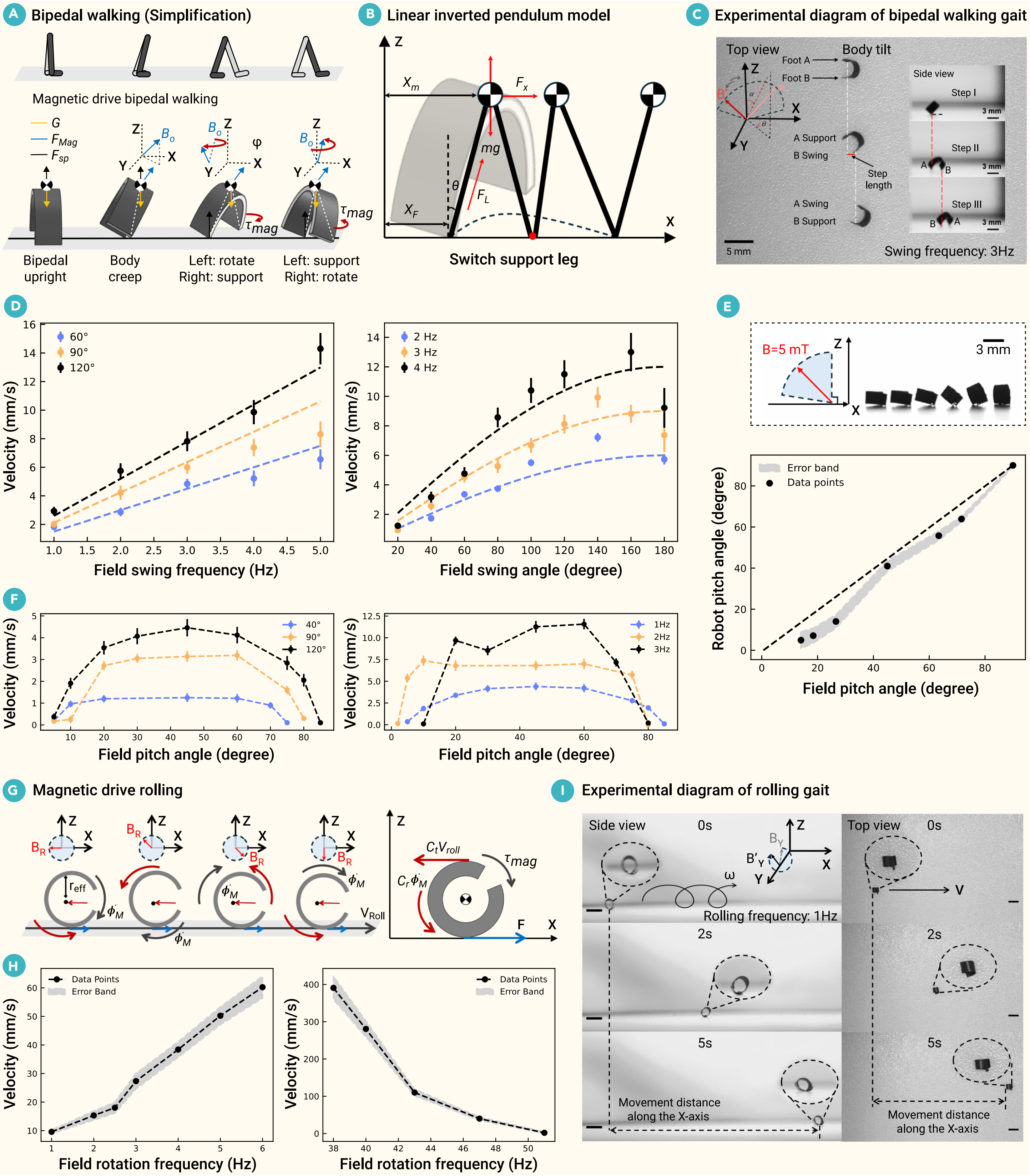

Figure 3. Dynamics of bipedal walking and wheel rolling gaits (A–F) Analysis of the bipedal walking gait. (A) A schematic and force analysis of the gait, driven by a swinging magnetic field, is modeled as a continuous linear inverted pendulum (B). (C) Experimental visualization of the walking sequence. (D–F) Comparisons between theoretical predictions (dotted lines) and experimental data show the relationship between travel speed and magnetic field swing frequency/amplitude (D), the correlation between the magnetic field and millirobot pitch angles (E), and the effect of pitch angle on travel speed (F). (G–I) Analysis of the wheel rolling gait. (G) A force analysis of the gait, driven by a rotating magnetic field, is shown alongside an experimental visualization (I). (H) The relationship between average rolling speed and the magnetic field's rotation frequency is plotted. In (D)–(F) and (H), error bars or bands represent SD. The inset in (I) shows an enlarged view of the millirobot. Scale bar, 4 mm.

Based on the linear inverted pendulum theory, the vertical component of the magnetic force is equal to and opposite gravity, and the motion of the center of mass in the horizontal direction is determined by the horizontal component of the magnetic force. The force along the leg is

$$\boldsymbol{F_L} = m\boldsymbol{g} \Big/ \cos\theta = m\boldsymbol{g}\frac{L}{z_F - z_m}, \quad \text{(Equation 16)}$$

where $\theta$is the angle between the swinging leg and the vertical direction.

Therefore,

$$\boldsymbol{F_x} = \boldsymbol{F_L}\sin\theta. \quad \text{(Equation 17)}$$

It is noted that when the driving frequency exceeds the natural frequency of the system, the millirobot is unable to follow the changes in the magnetic field and consequently loses synchronization. The natural frequency is determined by the inverted pendulum model, given by $f_c = \frac{1}{2\pi}\left(\frac{g}{L\cos\theta}\right)^{1/2}$. Additionally, it is also observed that out-of-step motion occurs when the swing angle causes the supporting leg to slide, which happens when $\boldsymbol{F_x} \geq \mu\boldsymbol{F_z}$. The critical value is determined by the static friction coefficient μ. Thus, the maximum swing angle is given by $\theta_{max} = arctan(\mu)$.

When the millirobot is moving at a low speed, based on the geometric kinematic relationship, the swing step d is related to the width D of the millirobot and the actual yaw angle φ, which can be approximated as

$$d = D\sin\frac{min(\varphi, 2\theta_{max})}{2}. \quad \text{(Equation 18)}$$

The average speed v of continuous travel can be approximated a:

$$v = 2k_v df = 2k_v\, min(f, f_c)D\sin\frac{min(\varphi, 2\theta_{max})}{2}, \quad \text{(Equation 19)}$$

where k_v is a speed gain that can be estimated based on experimental data.

The theoretical model was validated through a series of experiments, with the results presented in Figure 3 and Video S1. Unless otherwise stated, the magnetic field pitch angle was set to 45°. Three states of bipedal walking were depicted: the gait features a forward lean caused by the pitch angle of the field and a side-to-side sway driven by a 3 Hz horizontal oscillation (Figure 3C). As predicted by the theoretical model, the average walking speed exhibited a nearly linear positive relationship with the magnetic field's swing frequency and a positive, non-linear correlation with its swing angle amplitude. Experimental data closely matched these theoretical trends at lower frequencies and amplitudes (Figure 3D). However, as these parameters increased, a growing deviation was observed. Beyond a certain threshold, a "step-out" phenomenon occurred where the millirobot could not maintain synchronization with the rapidly changing magnetic field, resulting in a sharp decrease in speed. The millirobot's actual pitch angle was found to be consistent with the theoretical derivations (Equation 12), closely following the applied field's pitch angle (Figure 3E). However, postural instability and vertical shaking were observed for field pitch angles below 10°, which was attributed to the millirobot's uneven mass distribution. While theory predicts that pitch angle should not affect average speed (Equation 19), experiments revealed a stable velocity plateau for angles between 10° and 70° under low-frequency actuation (Figure 3F). Deviations from this plateau occurred at low angles (<10°) due to postural instability and at high frequencies due to the aforementioned step-out effects. As the pitch angle approached 90° (an upright state), the speed declined sharply. This is because the point-contact assumption of the model was violated; the increased contact area introduced significant, anisotropic friction, impeding motion. The steering characteristics were also quantitatively evaluated (Figure S2A). The millirobot's actual deflection angle was found to consistently overshoot the deflection angle of the applied magnetic field (Figure S2B). This effect, which becomes more pronounced at higher frequencies, is attributed to the millirobot's inertia.

Rolling gait of the wheeled millirobot. Driven by a rotating magnetic field, the wheeled millirobot achieves a movement similar to wheel rolling (Figure 3G). The rolling gait driven by the magnetic field has a speed constraint, known as the step-out frequency. Below this critical frequency, the millirobot motion is synchronized with the external field, characterized by a constant step size and a rolling frequency equal to that of the rotating magnetic field.

Mathematically, the rotating magnetic field $\boldsymbol{B_R}$ described above can be represented as

$$\boldsymbol{B_R}(t) = B\begin{pmatrix}\cos(2\pi ft)\\ 0\\ -\sin(2\pi ft)\end{pmatrix}, \quad \text{(Equation 20)}$$

where f represents the frequency of the $\boldsymbol{B_R}$.

As $\boldsymbol{B_R}$ starts to rotate clockwise, a rigid-body magnetic torque, τ_{mag}, is induced onto the millirobot, as the millirobot's $\mathbf{M}$ tends to align with $\boldsymbol{B_R}$. This torque makes the millirobot roll.

$$\tau_{mag} = \mathbf{M} \times \boldsymbol{B_R} = MB_R\sin(2\pi f - \phi_M), \quad \text{(Equation 21)}$$

where $\mathbf{M}$ is the net magnetic moment of the millirobot, f is the frequency of the rotating magnetic field, and ϕ_M is the millirobot rotational displacement.

The motion equation of the millirobot is as follows:

$$\mathbf{F} - C_t\boldsymbol{V_{roll}} = m\dot{\boldsymbol{V}}_{\boldsymbol{Roll}}, \ and \quad \text{(Equation 22)}$$

$$MB_R\sin(2\pi f - \phi_M) - C_r\dot{\boldsymbol{\phi}}_{\boldsymbol{M}} = I\ddot{\boldsymbol{\phi}}_{\boldsymbol{M}}, \quad \text{(Equation 23)}$$

where F is the thrust generated by the ground, m is the mass of the robot, I is the moment of inertia, and C_t and C_r are the translational and rotational damping coefficients, respectively.

For the millirobot driven by a rotating magnetic field, the angle between the driving field and the angular displacement of the millirobot tends to be constant under the steady-state velocity; that is, ϕ_M is a constant. When the magnetic moment is equal to the drag moment, the limit value at the maximum angular velocity of the millirobot can be obtained:

$$\omega_{\text{step-out}} = \frac{MB_R}{C_r}. \quad \text{(Equation 24)}$$

Under the step-out frequency, $2\pi f - \phi_M = k$, where k is a constant and does not change with time once the microrobot reaches its steady-state speed. By differentiating this expression in terms of time, this implies that

$$\dot{\boldsymbol{\phi}}_{\boldsymbol{M}} = 2\pi f. \quad \text{(Equation 25)}$$

Based on Equation 25 and the no-slip condition, the tumbling motion speed is

$$V_{roll} = 2\pi r_{eff} f. \quad \text{(Equation 26)}$$

To validate the theoretical model, the millirobot's frequency response was characterized experimentally (Video S1). The step-out frequency was determined to be approximately 38 Hz. As predicted, the millirobot's rolling speed showed a linear proportionality to the drive frequency up to this threshold (Figure 3H, left). Beyond the step-out frequency, the robot was unable to keep pace with the magnetic field changes, leading to a sharp decline in speed (Figure 3H, right). A key finding was that this rolling locomotion was significantly faster than the walking gait. The stable rolling motion depicted in Figure 3I, for example, was conducted at 1 Hz.

Motion controller

After determining the body shape and motion mode of the millirobot, the constraint conditions of the magnetic field control parameters are established based on the above motion models. Subsequently, the millirobot can be treated as a centroid model for kinematic analysis. The simplified nonholonomic system we consider describes the motion of a millirobot as follows:

$$\begin{cases}\dot{\boldsymbol{x}}(t) = v(f(t), A(t))\cos\theta(t)\\ \dot{\boldsymbol{y}}(t) = v(f(t), A(t))\sin\theta(t)\\ \dot{\boldsymbol{\theta}}(\tau) = w(t)\end{cases}, \quad \text{(Equation 27)}$$

where (x,y) is the global position of the millirobot, θ is the steering angle of the millirobot, and v and w are the millirobot velocity and angular velocity, respectively. The velocity is a function of the frequency $f(t)$ and amplitude $A(t)$ of the magnetic field (see Equations 19 and 26). Once the motion mode of the millirobot is determined, small variations in humidity or temperature affect only the leg width D of the legged morphology or the wheel radius r_{eff} of the wheeled morphology. These changes primarily influence the movement speed of the millirobot, while any disturbances to the movement trajectory are mitigated by the feedback controller.

Based on the motion model, the key input (magnetic field parameters)-output (speed and direction) relationships are abstracted to design the path-following controller of the polymorphic millirobot (Figure 4A). The dual-layer motion controller simultaneously received parallel control commands for high-level and low-level control. The high-level control system outputted the motion direction (swinging axis or rotating axis) to eliminate path-following errors, while the low-level control system implemented precise magnetic field output through current closed-loop control. Environmental and positional information is fed back through visual images.

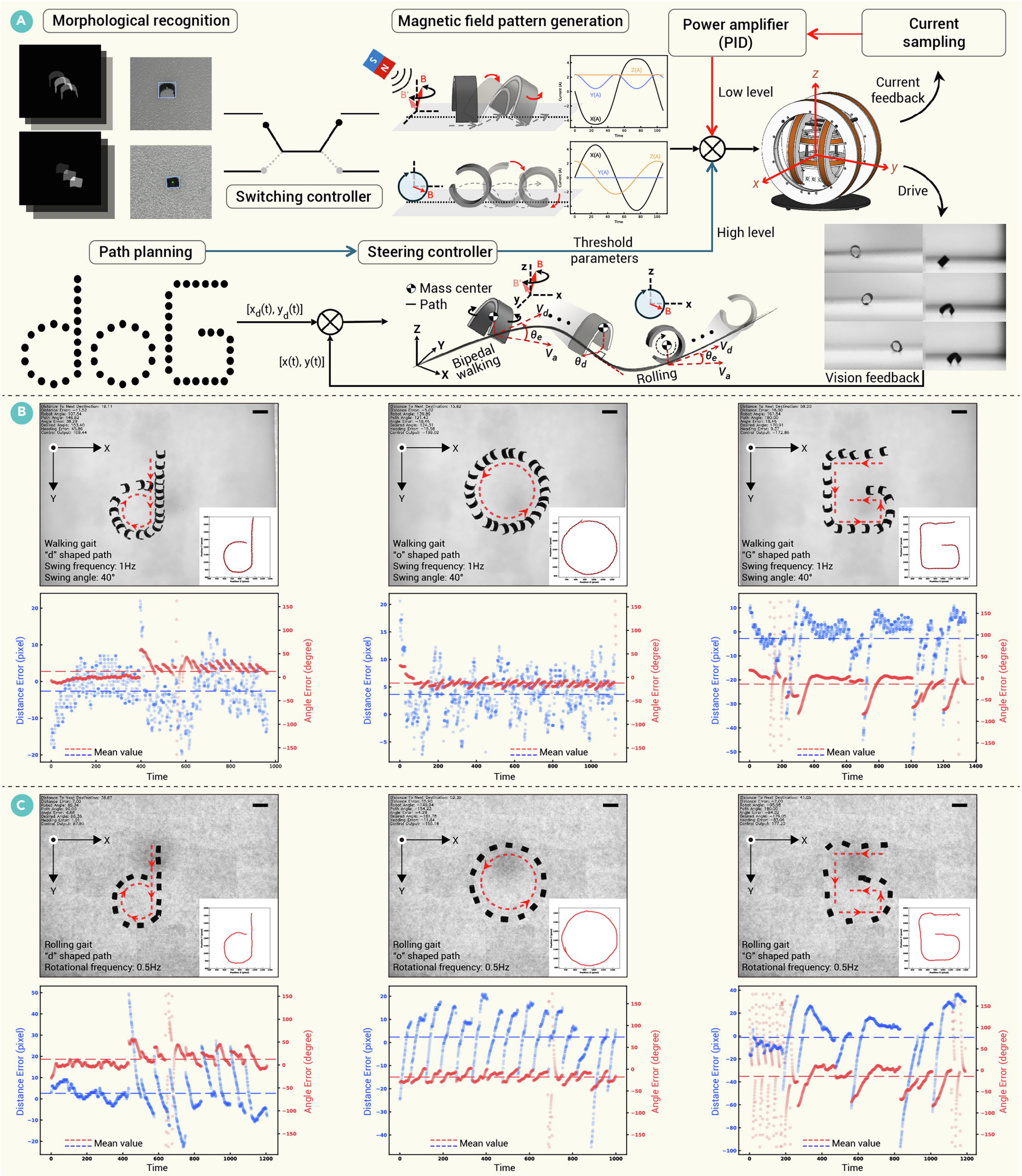

Figure 4. Path-following control of the polymorphic millirobot (A) Block diagram of the hierarchical path-following controller. (B and C) The legged millirobot follows a predefined doG trajectory using a bipedal walking gait (B), while the wheeled millirobot follows the same path using a rolling gait (C). Associated graphs for each image display the time evolution of angle and distance errors, where the *x* axis represents the sampling sequence. Scale bar, 5 mm.

For an arbitrary path, it can be characterized as a set of points $\mathcal{P} = \{P_1, P_2, \ldots, P_i, \ldots, P_j\}$. In path-following problems, the objective of control is to adjust the guidance law in such a way that the error of distance and angle becomes less than or equal to δ (where δ represents a very small positive value). The angle error is defined as the direction of the millirobot motion and the line direction of the nearest desired path segment. The distance error is defined as the vertical distance from the millirobot center of mass to the desired path.

Table 1. Distance error of path following (mm)

Experimental type	Error type	Open loop	PP	PID	MPC	Our method
The legged/wheeled millirobot follows the “d” path	RMS	2.32/3.99	1.88/1.9	1.94/2.35	0.72/1.05	0.3/0.38
	max	5.95/7.7	3.15/4.21	5.44/6.77	1.57/1.99	1.23/2.77
The legged/wheeled millirobot follows the “o” path	RMS	3.3/4.52	2.24/3.45	2.45/3.11	0.89/1.77	0.24/0.48
	max	6.72/9.13	4.75/5.13	5.6/6.54	1.86/3.01	1.15/2.52
The legged/wheeled millirobot follows the “G” path	RMS	3.87/5.96	2.73/4.74	3.32/4.62	0.63/1.38	0.37/0.84
	max	5.67/1	4.98/6.03	5.77/8.01	1.85/4.98	2.8/5.04

RMS, root mean square; max, maximum.

Thus, error vectors are defined as

$$E_\theta = \cos^{-1}\frac{\boldsymbol{L}_{P_{i+1}}^{P_i}\cdot\boldsymbol{L}_{C_t}^{C_{t-1}}}{\left\|\boldsymbol{L}_{P_{i+1}}^{P_i}\right\|\left\|\boldsymbol{L}_{C_t}^{C_{t-1}}\right\|},\ and \qquad \text{(Equation 28)}$$

$$\boldsymbol{E_d} = \frac{\boldsymbol{L}_{P_{i+1}}^{P_i}\times\boldsymbol{L}_{P_i}^{C_t}}{\left\|\boldsymbol{L}_{P_{i+1}}^{P_i}\right\|^2}\boldsymbol{L}_{P_{i+1}}^{P_i} - \boldsymbol{L}_{P_i}^{D_K}, \qquad \text{(Equation 29)}$$

where $\boldsymbol{L}_{P_{i+1}}^{P_i}$ is a path vector from P_i to P_{i+1}, C indicate the barycenter of the millirobot, $\boldsymbol{L}_{C_t}^{C_{t-1}}$ is a motion vector from times $(t-1)$ to t, and D_K is the projection of C_t onto the segment $\boldsymbol{L}_{P_{K+1}}^{P_K}$.

Based on the control positive gain k, we design the guidance law $\boldsymbol{n_d}$,

$$\boldsymbol{n_d} = sign(\boldsymbol{E_d})\times\left(\frac{f(\boldsymbol{E_d})}{k+f(\boldsymbol{E_d})}\cdot\frac{\boldsymbol{E_d}}{\|\boldsymbol{E_d}\|}+\frac{k}{k+f(\boldsymbol{E_d})}\cdot\frac{\boldsymbol{L}_{P_{i+1}}^{P_i}}{\left\|\boldsymbol{L}_{P_{i+1}}^{P_i}\right\|}\right), \qquad \text{(Equation 30)}$$

where $f(\boldsymbol{E_d}) = \frac{\|\boldsymbol{E_d}\|}{1+\gamma\|\boldsymbol{E_d}\|}$ is a non-linear scaling function used to smooth out distance errors and $\gamma > 0$ is the adjustment factor. Increasing γ allows the robot to approach the desired path more smoothly, whereas decreasing γ enables the robot to turn toward the desired path more quickly. A larger value of k inclines the millirobot to move more along the direction of the current path segment, while a smaller value of k encourages the millirobot to attempt more actively to eliminate the distance error.

This study addresses the path-following capabilities of millirobots by remotely manipulating their propulsive direction. This is achieved by adjusting the yaw angle θ of the principal axis of the magnetic field (both rotating/oscillating magnetic fields) while keeping the frequency and amplitude (as well as the oscillation amplitude) constant. To facilitate effective tracking under these conditions, a proportional-integral controller is designed to dynamically adjust the direction of the magnetic field spindle, thereby reducing the current tracking error and eliminating steady-state tracking errors during operation.

Then, the control output $\Delta\theta$ is defined as

$$\theta_e = atan\,2\left(\boldsymbol{L}_{C_t}^{C_{t-1}},\boldsymbol{n_d}\right), \qquad \text{(Equation 31)}$$

$$\tilde{\theta} = \begin{cases} \theta_e + 2\pi, \text{if } \theta_e \in [-2\pi, -\pi] \\ \theta_e, \text{if } \theta_e \in (-\pi, \pi] \\ \theta_e - 2\pi, \text{if } \theta_e \in (\pi, 2\pi] \end{cases},and \qquad \text{(Equation 32)}$$

$$\Delta\theta = \mathrm{Sat}\left(k_p\times\tilde{\theta}+k_i\times\sum_{m=1}^{n}\tilde{\theta}_e^m, 20\right), \qquad \text{(Equation 33)}$$

where θ_e is the misalignment between the current propulsion and the guidance law and k_p and k_i denote positive proportional and integral gains, respectively.

RESULTS AND DISCUSSION

Magnetic drive system

A home-built 3D Helmholtz coil system was used to generate the required magnetic fields, producing a peak intensity of 15 mT with less than 1% non-uniformity within a 100-mm-diameter central workspace (Figure S3). Further details on this system, whose parameters are listed in Table S1, have been previously published.[49,50] The control architecture consisted of a host PC that generated real-time current commands and a microcontroller that produced corresponding pulse width modulation (PWM) signals. These signals regulated three motor drives to supply current to the coils. Industrial cameras provided continuous real-time tracking of the millirobot.

Based on the assumption that the magnitude of the magnetic field in the region of interest changes linearly, at any given point P in the workspace, the magnetic field can be expressed as the sum of the three coil contributions.

$$B(P) = \sum_{i=1}^{n}\tilde{B}_i(P)I_i = \tilde{\boldsymbol{B}}(P)\boldsymbol{I}, \qquad \text{(Equation 34)}$$

where n indicates the number of coils, $\tilde{B}_i(P) \in \mathbb{R}^{3\times1}$ is a matrix that depends on the measuring point P, and $I \in \mathbb{R}^{3\times1}$ denotes the control current in each coil. The simulation results of the dynamic magnetic field are shown in Figures S4 and S5.

Path-following control

To determine the millirobot's actual direction of motion, its gear-shaped trajectory, a product of its approximate symmetry, was linearly fitted. This real-time fitting was performed using n_k centroid points, where n represents the current time step and k is the number of points used in the summation. The real-time centroid points and the resulting fitted trajectories are presented in Video S2. To validate the control strategy, both the legged and wheeled millirobots were tasked with following a path shaped like the letters “doG.” In these trials, both the angle and distance errors of motion converged to near zero. Image sequences, position coordinates, and the corresponding error analysis were recorded (Figures 4B and 4C; Video S2). A comparative analysis demonstrated that the proposed control method achieved significantly improved path-following accuracy over open-loop control, pure pursuit (PP), traditional proportional-integral-derivative (PID) control, and model predictive control (MPC) (Table 1).

Traversing multiple terrains

The millirobot's distinct locomotion modes proved to be complementary, enabling it to navigate a variety of challenging terrains where a single mode would fail (Video S3). Specifically, the legged walking gait enabled the millirobot to stably ascend a 17.7° slope (Figures 5A and 5B). This mode also allowed it to successfully cross a 2.6 mm gap (Figures 5C and 5D). In contrast, the wheeled configuration was unable to cross the same gap, as its width exceeded the robot's diameter in its rolling form, causing it to fall in (Figure S6). The wheeled mode excelled at smoothly and efficiently traversing curved surfaces (with peak heights of 3 and 4 mm) and ascending a flight of stairs (1 mm step height) (Figures 5E–5H). The legged millirobot, however, struggled on these terrains; it was unable to maintain stability on the steep inclines of the curved surface and could not lift its legs high enough to clear the steps.

Bimodal transformation and navigation

The millirobot's ability to perform a bimodal transformation while in motion was evaluated (Figure S7A; Video S4). Initially, the millirobot adopted a C-shaped conformation, which enabled legged locomotion actuated by a 2 Hz swinging magnetic field (Figures S7B and S7C). Following an increase in ambient temperature, water evaporation from the millirobot's body induced a structural contraction, causing the C-shape to gradually close into a circular, wheel-like form (Figures S7D and S7E). Once the transformation into this wheeled configuration was complete, a 2 Hz rotating magnetic field was applied to achieve directional rolling motion (Figures S7F and S7G).

By leveraging this morphological transformation, the millirobot successfully navigated a narrow, unstructured channel formed by randomly scattered stones. The channel's narrowest point measured approximately 2 mm, demonstrating the practical application of its adaptive locomotion (Figure S8; Video S5).

www.the-innovation.org

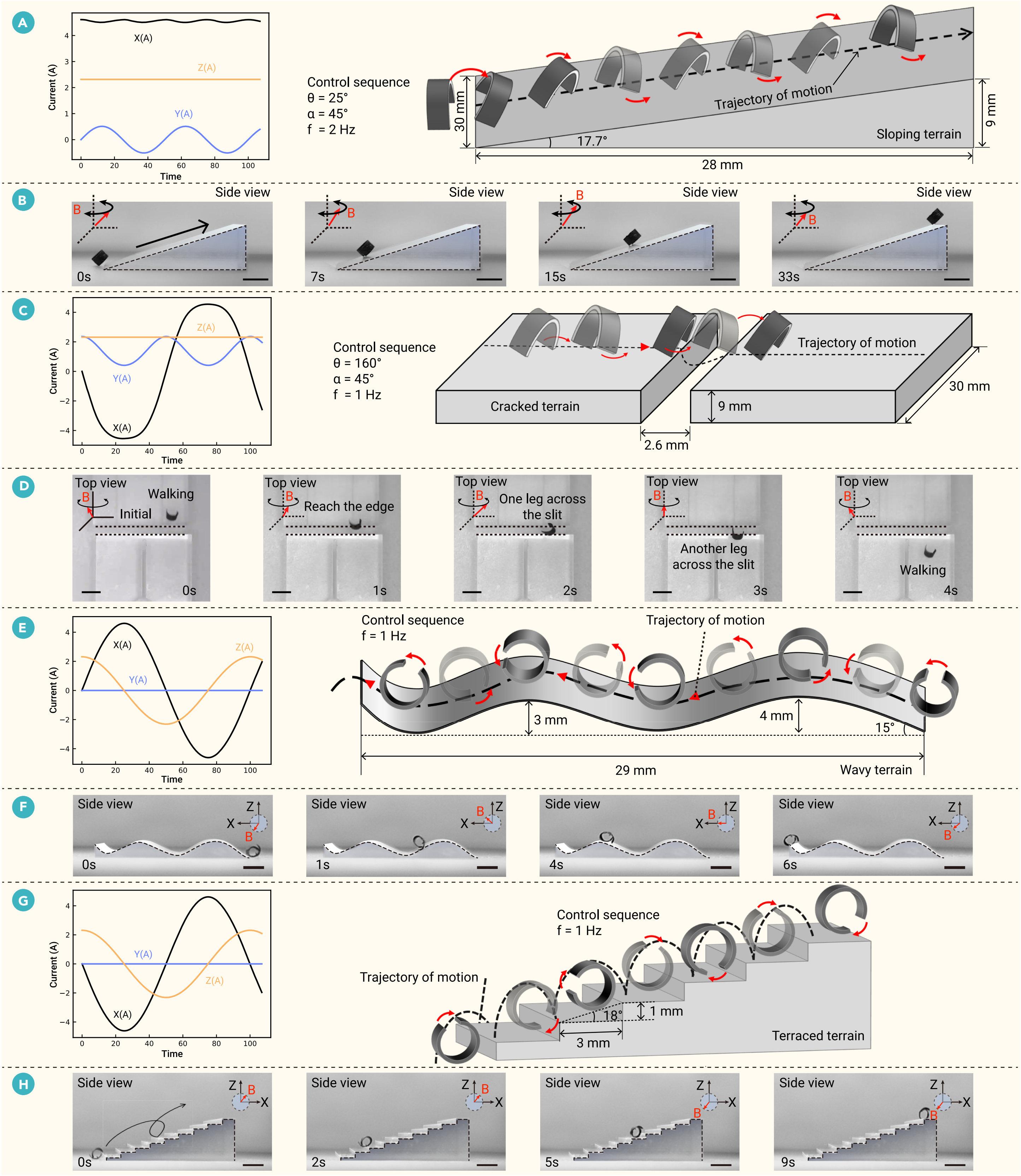

Figure 5. Multimodal locomotion of the millirobot across various terrains (A and B) Traversing a slope via bipedal walking: control signals (A) use a swinging magnetic field (45° pitch, 25° swing angle, 2 Hz frequency). The motion sequence is shown in (B). Scale bar: 5 mm. (C and D) Traversing a gap via bipedal walking: control signals (C) use a swinging magnetic field (45° pitch, 160° swing angle, 1 Hz frequency). The motion sequence is shown in (D). Scale bar: 5 mm. (E–H) Traversing curved relief and stairs via wheel rolling: control signals (E and G) use a rotating magnetic field at 1 Hz. The motion sequences for the curved relief (F) and stairs (H) are shown. Scale bar: 4 mm.

Task operation in complex environment

Leveraging its temperature- and humidity-responsive deformation, the 2.7 mg millirobot transported a 2.5-mm-diameter, 36 mg ball (Figures 6A and 6B; Video S6). A swinging magnetic field (1 Hz frequency, 45° pitch, 60° swing angle) propelled the legged millirobot toward the target cargo. Upon approach, it used magnetic fields to reposition itself and encircle

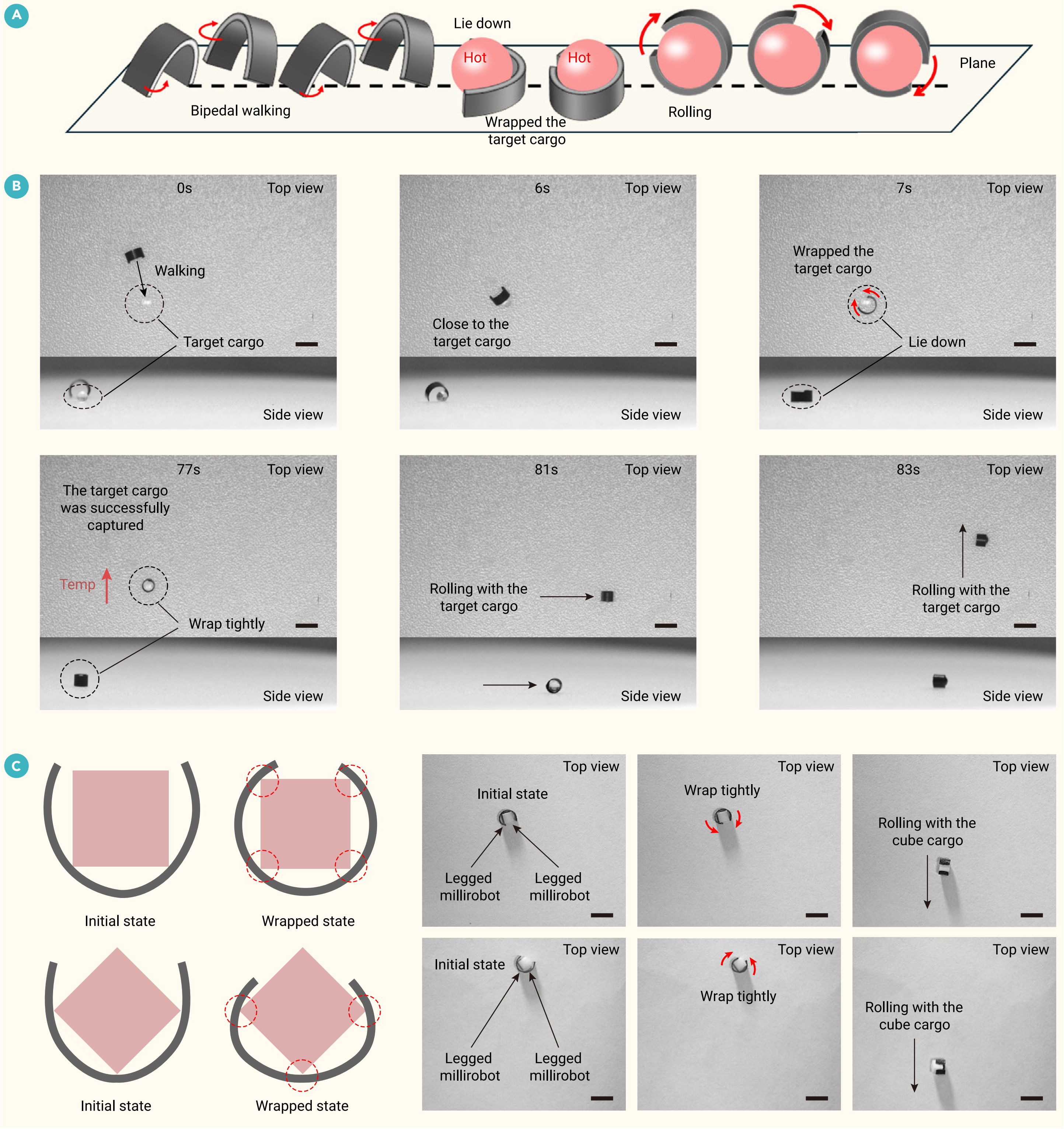

Figure 6. Cargo capture and transport by the wheel-legged polymorphic millirobot (A) Schematic of the cargo capture and transport process. (B) The millirobot walks to, automatically grasps (triggered by environmental changes), and transports the 2.5 mm spherical cargo. (C) Grasping and transporting a cube cargo by winding around it at three to four contact points. Scale bar: 4 mm.

the target. Subsequently, due to the higher temperature of the target cargo compared to the environment, the millirobot lost water and shrank until it completely wrapped the cargo. A rotating magnetic field (1 Hz) was then activated, and the wheeled millirobot rolled away, carrying the cargo. To further demonstrate its object manipulation capabilities, the millirobot also wrapped and transported a 2 mm, 18 mg cube, successfully securing it at three or four contact points during these tests (Figure 6C; Video S6).

The millirobot navigated four distinct terrains as shown on a single map (Figure 7; Video S7). It first ascended a 15° slope with a walking gait and then extended its stride to cross a 2 mm gap. After descending a 3.75 mm platform, the robot shortened its stride to maneuver in a confined space and approach the cargo. It then lowered itself to conform to the cargo's shape. An increase in ambient temperature caused the robot to contract, securing the cargo. Subsequently, the millirobot ascended five 1 mm steps and traversed a curved path with a 1.3 mm vertical depression

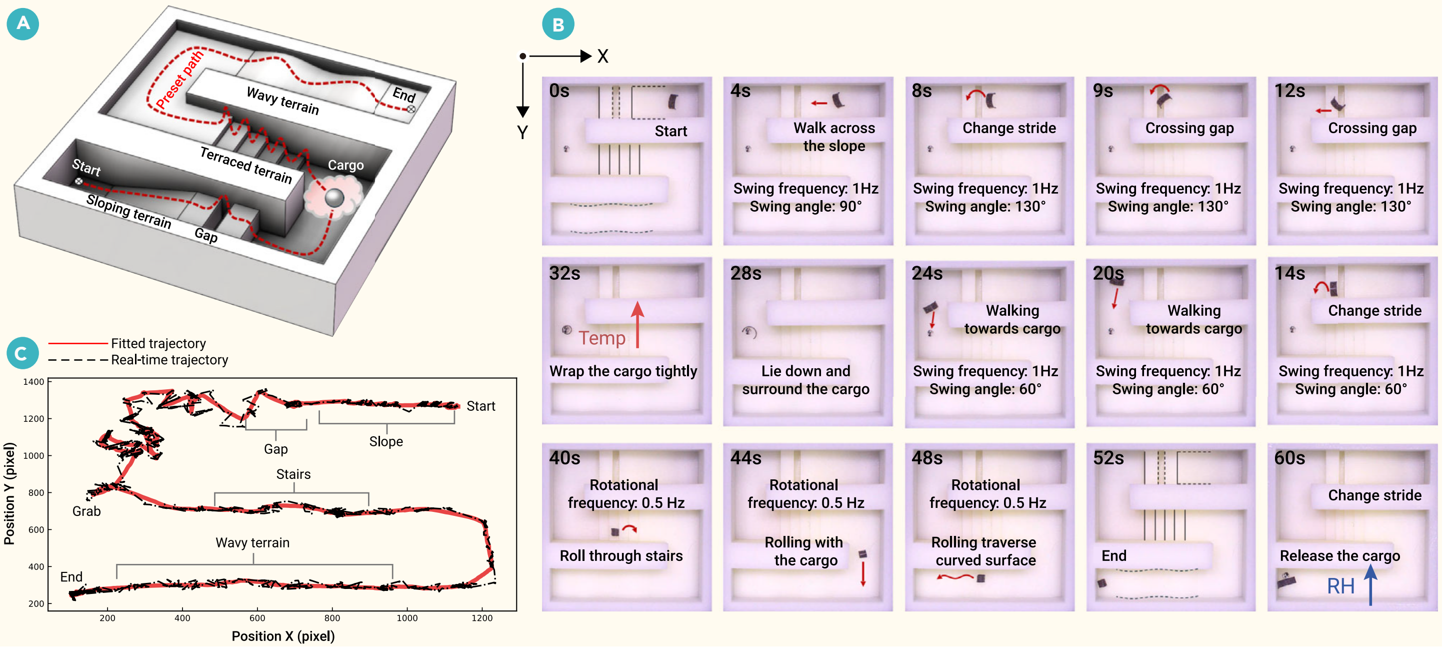

Figure 7. The wheel-legged polymorphic millirobot performs tasks in complex and narrow environments (A) Topographic map. (B) Experimental image sequence. Scale bar: 5 mm. (C) Motion trajectory diagram.

using a rolling gait. Finally, increased ambient humidity prompted the robot to expand and release the cargo.

CONCLUSION

This study proposed a polymorphous millirobot based on multi-source stimulus-responsive materials and designed matching motion modes according to morphological characteristics to achieve stable, multimodal, accurate motion control of the millirobot while operating in harmony with varied and unstructured environments. Our research solved the problems that a single motion mode has difficulty adapting to the changing terrain and environment and that guaranteeing the stability of all motion modes of a monomorphic millirobot is challenging. For the problem of multi-source excitation coupling, we decoupled deformation and motion response based on multi-layer shape design to achieve independent control of both. In addition, motion models under different body morphologies are established to provide guidance for robotized motion control of deformable materials. The motion experiments under different terrain environments confirmed the high environmental adaptability, the accurate path-following experiments confirmed the high controllability, and the targeted cargo sampling and transportation experiments confirmed the effectiveness of the polymorphic cooperative task. Table S2 presents a comparison of the performance characteristics between the robot reported in this study and existing soft robots, which underscores the unique strengths of our design, including its superior adaptability, competitive payload capacity, and enhanced controllability. Our millirobot structure design concept and motion control method provided new ideas for the manufacture and application of millirobots based on deformable materials and is expected to be applied in precision operations. Certainly, wear and material degradation could potentially impact the millirobot's functionality over even longer periods and may introduce certain limitations. For instance, prolonged use in harsh environments or under extreme conditions might accelerate material wear, which might compromise the millirobot's ability to contract and deform symmetrically, leading to asymmetry in the lengths of the legs of the legged millirobot or the inability of the wheeled millirobot to close properly. We recognize that further research is needed to enhance the millirobot's robustness for long-term operations. Future work will focus on exploring advanced materials with higher wear resistance and developing adaptive control algorithms to compensate for any degradation in performance.

RESOURCE AVAILABILITY

Materials availability

This study did not generate new unique materials/reagents.

Data and code availability

Data are available from the corresponding author upon reasonable request.

FUNDING AND ACKNOWLEDGMENTS

This work is supported by the National Natural Science Foundation of China under grant numbers 62222305, 62088101, and U22A2064; the Beijing Natural Science Foundation under grant L242023; and the Fundamental Research Funds for the Central Universities under grants 2025CX01003 and 2024CX06008. The funders had no role in the study design, data collection and analysis, decision to publish, or preparation of the manuscript.

AUTHOR CONTRIBUTIONS

S.Z., R.N., Z.Z., and Y.H. conceived the idea and designed the research. S.Z. and R.N. constructed the experimental platform. S.Z., Q.S., Q.H., and H.W. performed the research and analyzed the data. S.Z. and Z.Z. formulated and implemented the computational model. Z.Z. and H.W. supervised the research. The manuscript was written by S.Z., R.N., Z.Z., Y.H., Q.S., Q.H., T.F., and H.W. with contributions from all authors during discussions and manuscript revisions. All authors contributed to the manuscript and approved the final version.

DECLARATION OF INTERESTS

The authors declare that they have no conflicts of interest.

SUPPLEMENTAL INFORMATION

It can be found online at https://doi.org/10.1016/j.xinn.2025.101146.

REFERENCES

1. Zhang, Z., Wang, X., Liu, J. et al. (2019). Robotic micromanipulation: Fundamentals and applications. *Annu. Rev. Control Robot. Auton. Syst.* **2**:181–203. DOI:10.1146/annurev-control-053018-023755
2. Wang, H., Liu, J., Nie, R. et al. (2025). Bio-integrated microrobots: fabrication, actuation and biomedical applications. *SmartBot* **1**:e12007. DOI:10.1002/smb2.12007
3. Wang, Q., Zhao, F., Wang, B. et al. (2025). Untethered miniature robots for minimally invasive thrombus treatment: From bench to clinical trials. *Innovation* **6**:100874. DOI:10.1016/j.xinn.2025.100874
4. Khalil, I.S.M., Klingner, A., Hamed, Y. et al. (2020). Controlled noncontact manipulation of nonmagnetic untethered microbeads orbiting two-tailed soft microrobot. *IEEE Trans. Robot.* **36**:1320–1332. DOI:10.1109/TRO.2020.2990768
5. Wang, X., Li, S., Chang, J.C. et al. (2024). Multimodal locomotion ultra-thin soft robots for exploration of narrow spaces. *Nat. Commun.* **15**:6296. DOI:10.1038/s41467-024-50598-1
6. Hou, Y., Zhong, S., Zheng, Z. et al. (2025). Magnetic shaftless propeller millirobot with multi-modal motion for small-scale fluidic manipulation. *Cyborg Bionic Syst.* **6**:0235. DOI:10.34133/cbsystems.0235

7. Peng, Y., Liu, X., Chan, K.F. et al. (2025). Robotic-assisted intravascular embolization: progress and future perspectives. *SmartBot* **1**:e12009. DOI:10.1002/smb2.12009
8. Liu, J., Qin, S., Hou, Y. et al. (2025). Data-driven adaptive control of multiple active micromotors with optoelectronic guidance for independent transport and cargo delivery. *IEEE ASME Trans. Mechatron.* **30**:2919–2927. DOI:10.1109/TMECH.2025.3572199
9. Wu, Y., Dong, X., Kim, J.-k. et al. (2022). Wireless soft millirobots for climbing three-dimensional surfaces in confined spaces. *Sci. Adv.* **8**:eabn3431. DOI:10.1126/sciadv.abn3431
10. Gong, S., Fang, F., Yi, Z. et al. (2024). An intelligent spinal soft robot with self-sensing adaptability. *Innovation* **5**:100640. DOI:10.1016/j.xinn.2024.100640
11. Hu, W., Lum, G.Z., Mastrangeli, M. et al. (2018). Small-scale soft-bodied robot with multimodal locomotion. *Nature* **554**:81–85. DOI:10.1038/nature25443
12. Ze, Q., Wu, S., Dai, J. et al. (2022). Spinning-enabled wireless amphibious origami millirobot. *Nat. Commun.* **13**:3118. DOI:10.1038/s41467-022-30802-w
13. Zhao, J., Xin, C., Zhu, J. et al. (2024). Insect-scale biped robots based on asymmetrical friction effect induced by magnetic torque. *Adv. Mater.* **36**:2312655. DOI:10.1002/adma.202312655
14. Asadi, F. and Hurmuzlu, Y. (2024). Motion planning for multiple heterogeneous magnetic robots under global input. *IEEE Trans. Robot.* **40**:697–713. DOI:10.1109/TRO.2023.3339529
15. Deng, Y., Paskert, A., Zhang, Z. et al. (2023). An acoustically controlled helical microrobot. *Sci. Adv.* **9**:eadh5260. DOI:10.1126/sciadv.adh5260
16. Hou, C., Wang, K., Wang, S. et al. (2024). Artificial bacteria flagella with micro-structured soft-magnetic teeth. *IEEE Trans. Robot.* **40**:4719–4732. DOI:10.1109/TRO.2024.3463482
17. Wang, Y., Du, X., Zhang, H. et al. (2023). Amphibious miniature soft jumping robot with on-demand in-flight maneuver. *Adv. Sci.* **10**:e2207493. DOI:10.1002/advs.202207493
18. Ren, Z., Zhang, R., Soon, R.H. et al. (2021). Soft-bodied adaptive multimodal locomotion strategies in fluid-filled confined spaces. *Sci. Adv.* **7**:eabh2022. DOI:10.1126/sciadv.abh2022
19. Zhong, S., Xin, Z., Hou, Y. et al. (2024). Double-Modal Locomotion of a Hydrogel Ultra-Soft Magnetic Miniature Robot with Switchable Forms. *Cyborg Bionic Syst.* **6**:0077. DOI:10.34133/cbsystems.0077
20. Lu, H., Zhang, M., Yang, Y. et al. (2018). A bioinspired multilegged soft millirobot that functions in both dry and wet conditions. *Nat. Commun.* **9**:3944. DOI:10.1038/s41467-018-06491-9
21. Huang, H., Feng, Y., Yang, X. et al. (2022). An insect-inspired terrains-adaptive soft millirobot with multimodal locomotion and transportation capability. *Micromachines* **13**:1578. DOI:10.3390/mi13101578
22. Xiang, Y., Liu, R., Wei, Z. et al. (2024). MINRob: A large force-outputting miniature robot based on a triple-magnet system. *IEEE Trans. Robot.* **40**:3127–3145. DOI:10.1109/TRO.2024.3410096
23. Shin, B., Ha, J., Lee, M. et al. (2018). Hygrobot: A self-locomotive ratcheted actuator powered by environmental humidity. *Sci. Robot.* **3**:eaar2629. DOI:10.1126/scirobotics.aar2629
24. Amjadi, M. and Sitti, M. (2016). High-performance multiresponsive paper actuators. *ACS Nano* **10**:10202–10210. DOI:10.1021/Acsnano.6b05545
25. Yang, X., Lan, L., Pan, X. et al. (2023). Bioinspired soft robots based on organic polymer-crystal hybrid materials with response to temperature and humidity. *Nat. Commun.* **14**:2287. DOI:10.1038/s41467-023-37964-1
26. Villeda-Hernandez, M., Baker, B.C., Romero, C. et al. (2023). Chemically driven oscillating soft pneumatic actuation. *Soft Robot.* **10**:1159–1170. DOI:10.1089/soro.2022.0168
27. Lee, J.A., Li, N., Haines, C.S. et al. (2017). Electrochemically powered, energy-conserving carbon nanotube artificial muscles. *Adv. Mater.* **29**:1700870. DOI:10.1002/adma.201700870
28. Kaynak, M., Dirix, P. and Sakar, M.S. (2020). Addressable acoustic actuation of 3D printed soft robotic microsystems. *Adv. Sci.* **7**:2001120. DOI:10.1002/advs.202001120
29. Lu, X., Zhao, K., Liu, W. et al. (2019). A human microrobot interface based on acoustic manipulation. *ACS Nano* **13**:11443–11452. DOI:10.1021/acsnano.9b04930
30. Lan, R., Sun, J., Shen, C. et al. (2020). Light-driven liquid crystalline networks and soft actuators with degree-of-freedom-controlled molecular motors. *Adv. Funct. Mater.* **30**:2000252. DOI:10.1002/adfm.202000252
31. Kim, M., Yu, A., Kim, D. et al. (2023). Multi-agent control of laser-guided shape-memory alloy microrobots. *Adv. Funct. Mater.* **33**:2304937. DOI:10.1002/adfm.202304937
32. Soon, R.H., Yin, Z., Dogan, M.A. et al. (2023). Pangolin-inspired untethered magnetic robot for on-demand biomedical heating applications. *Nat. Commun.* **14**:3320. DOI:10.1038/s41467-023-38689-x
33. Cloudsley-Thompson, J.L. (1975). Adaptations of Arthropoda to arid environments. *Annu. Rev. Entomol.* **20**:261–283. DOI:10.1146/annurev.en.20.010175.001401
34. Ballerio, A. and Smith, A.B.T. (2016). "Rolls up like Armadillo": Darwin's forgotten encounters with ceratocanthine beetles (Coleoptera: Hybosoridae). *Arch. Nat. Hist.* **43**:357–360. DOI:10.3366/anh.2016.0391
35. Bellini, L., Pagani, G., Mollo, A. et al. (2019). Evaluation of alfaxalone and dexmedetomidine for intramuscular restraint in European hedgehogs (Erinaceus europaeus). *Vet. Rec.* **185**:145. DOI:10.1136/vr.105231
36. Lu, Y., Ballerio, A., Wang, S. et al. (2022). The evolution of conglobation in Ceratocanthinae. *Commun. Biol.* **5**:777. DOI:10.1038/s42003-022-03685-2
37. Feng, W., He, Q. and Zhang, L. (2025). Embedded physical intelligence in liquid crystalline polymer actuators and robots. *Adv. Mater.* **37**:2312313. DOI:10.1002/adma.202312313
38. Chi, Y., Li, Y., Zhao, Y. et al. (2022). Bistable and multistable actuators for soft robots: Structures, materials, and functionalities. *Adv. Mater.* **34**:2110384. DOI:10.1002/adma.202110384
39. Guo, S., Zhang, Y. and Tan, S.C. (2023). Device design and optimization of sorption-based atmospheric water harvesters. *Device* **1**:100099. DOI:10.1016/j.device.2023.100099
40. Zheng, Z., Han, J., Shi, Q. et al. (2024). Single-step precision programming of decoupled multiresponsive soft millirobots. *Proc. Natl. Acad. Sci. USA* **121**:e2320386121. DOI:10.1073/pnas.2320386121
41. Guo, S., Zhang, S., Li, H. et al. (2025). Precisely manipulating polymer chain interactions for multifunctional hydrogels. *Matter* **8**:101785. DOI:10.1016/j.matt.2024.06.024
42. Zheng, Z., Han, J., Demir, S.O. et al. (2023). Electrodeposited superhydrophilic-superhydrophobic composites for untethered multi-stimuli-responsive soft millirobots. *Adv. Sci.* **10**:2302409. DOI:10.1002/advs.202302409
43. Dong, Y., Wang, J., Guo, X. et al. (2019). Multi-stimuli-responsive programmable biomimetic actuator. *Nat. Commun.* **10**:4087. DOI:10.1038/s41467-019-12044-5
44. Li, X., Liu, J., Li, D. et al. (2021). Bioinspired multi-stimuli responsive actuators with synergistic color-and morphing-change abilities. *Adv. Sci.* **8**:2101295. DOI:10.1002/advs.202101295
45. Dong, Y., Wang, L., Xia, N. et al. (2022). Untethered small-scale magnetic soft robot with programmable magnetization and integrated multifunctional modules. *Sci. Adv.* **8**:eabn8932. DOI:10.1126/sciadv.abn8932
46. Karacakol, A.C., Alapan, Y., Demir, S.O. et al. (2025). Data-driven design of shape-programmable magnetic soft materials. *Nat. Commun.* **16**:2946. DOI:10.1038/s41467-025-58091-z
47. Demir, S.O., Tiryaki, M.E., Karacakol, A.C. et al. (2024). Learning soft millirobot multimodal locomotion with sim-to-real transfer. *Adv. Sci.* **11**:2308881. DOI:10.1002/advs.202308881
48. Pietersen, D.W., Jansen, R., Swart, J. et al. (2020). Temminck's pangolin Smutsia temminckii. In Pangolins: Science, Society and Conservation, D.W.S. Challender, H.C. Nash, and C. Waterman, eds. (Academic Press), pp. 175–193. DOI:10.1016/B978-0-12-815507-3.00011-3
49. Zhong, S., Guo, S., Sun, T. et al. (2025). Paired interactions of magnetic millirobots in confined spaces through data-driven disturbance rejection control under global input. *IEEE ASME Trans. Mechatron.* DOI:10.1109/TMECH.2024.3521085
50. Xin, Z., Zhong, S., Wu, A. et al. (2025). Dynamic control of multimodal motion for bistable soft millirobots in complex environments. *IEEE Trans. Robot.* **41**:2662–2676. DOI:10.1109/TRO.2025.3551541

The Innovation, Volume 7

Supplemental Information

Magnetically controlled multimodal motion for environmentally adaptive soft millirobots with transformable wheel-leg morphology

Shihao Zhong, Ruhao Nie, Zhiqiang Zheng, Yaozhen Hou, Qing Shi, Qiang Huang, Toshio Fukuda, and Huaping Wang

This PDF file includes:

Note S1

Figures S1 to S8

Tables S1 to S2

Other supporting materials for this manuscript include the following:

Movies S1 to S7

SUPPLEMENTARY NOTE

S1. Fabrication process

PDMS-LIG-xerogel composite**:** First, LIG was generated onto the surface of polyimide (PI: 63μm, Kapton, Electron® Microscopy Sciences) tape by laser scribing (wavelength, 10.6 μm; beam size, ~120 μm, Universal Laser Systems, PLS6-150D). The parameters were set as follows: laser power (4 W), laser speed (260 mm/s), dots per inch (1,000), and pulse repetition rate (10.5 kHz). The uncured PDMS (base to curing agent weight ratio of 15:1) and NdFeB hard magnetic microparticles (Magnequench GmbH, MQP-15-7, diameter 5 μm) were mixed in a 1:1 weight ratio. Then, the PDMS mixed solution was poured onto the surface of LIG, and the PDMS-LIG double-layer structure was obtained after heating and curing (80°C, 6 hours). Due to the conductive properties of the LIG layer, alginate gel was electrodeposited on its surface. Prior to electrodeposition, the LIG-PDMS composite layer was treated with plasma for 10 minutes to enhance surface activity. Next, the sodium alginate solution was evenly spread over the top of the LIG side, and then vacuumed for 10 minutes to allow the sodium alginate to penetrate the surface of the LIG. During the electrodeposition process, the LIG layer served as the bottom electrode, while ITO glass was used as the top electrode. A 4 V DC voltage was applied for 120 seconds to initiate the alginate polymerization process. After electrodeposition, the uncured solution was carefully removed by washing the composite film with distilled water three times. The alginate gel was dried at room temperature for 48 hours to form a xerogel. Note that due to the porous structure of the LIG layer, the LIG layer formed a tight cross-linked network with the immersed hydrogel and PDMS solution and resulted in a solid three-layer nested structure after curing.

Laser Engraving-Based Xerogel Patterning**:** The 355 nm ultraviolet laser cutting system (LAJAMIN LASER) was employed to pattern the xerogel layer, enabling controlled stress distribution and programmable deformation. To selectively remove the xerogel layer, the optimized engraving parameters included a 10 A drive current, a scanning speed of 650 mm/s, and a pulse frequency of 50 kHz. Additionally, the ultraviolet laser cutting system was used to cut the required sheet shape for the three-layer composite structure. The optimized engraving parameters included a 10 A drive current, a scanning speed of 10 mm/s, and a pulse frequency of 20 kHz.

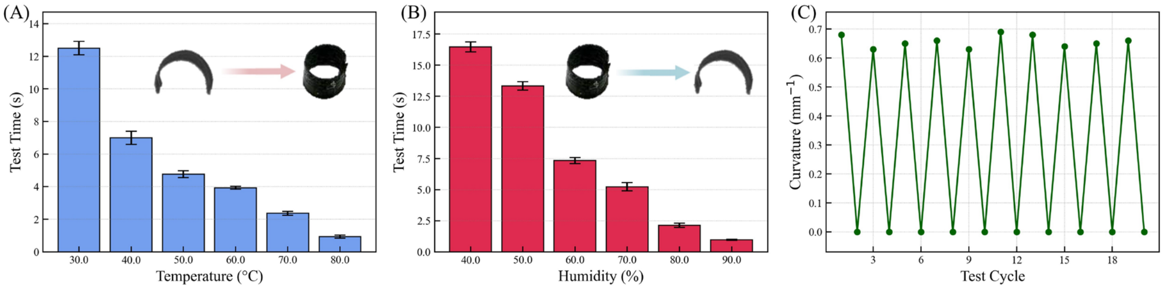

Figure S1. Response test under humidity/temperature excitation. (A) Transformation time from wheel to leg morphology under different temperature excitations. (B) Transformation time from leg to wheel morphology under different humidity excitations. (C) Results of wheel-leg shape transformation across multiple cycles in RH range 40% to 90% and temperature range 70°C to 30°C. Error bars represent SD.

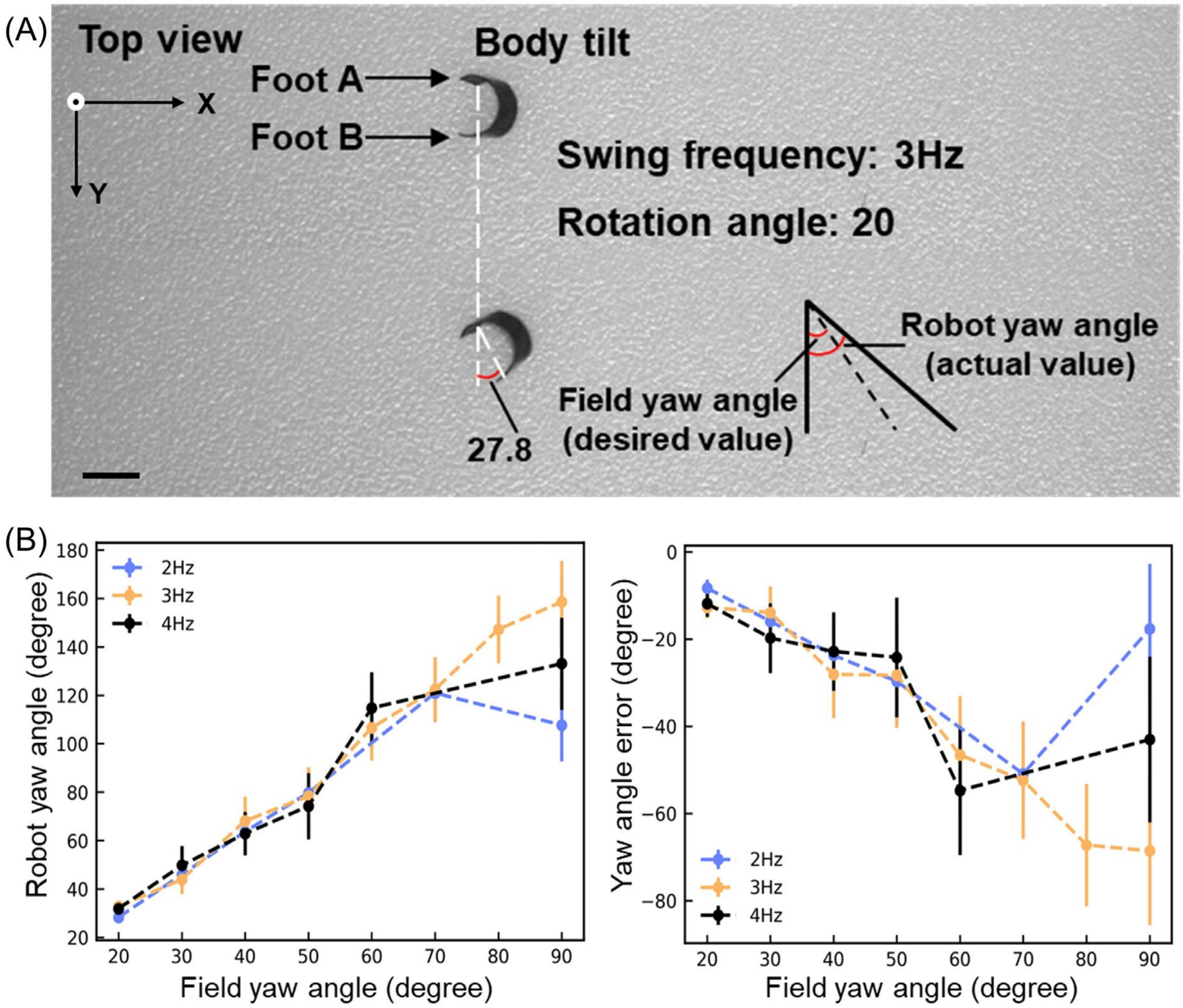

Figure S2. (A) Schematic diagram of magnetic field deflection angle and actual deflection angle of the millirobot. Scale bar: 3 mm. (B) Test results of the relationship between the magnetic field deflection angle and the actual deflection angle of the millirobot. Error bars represent SD.

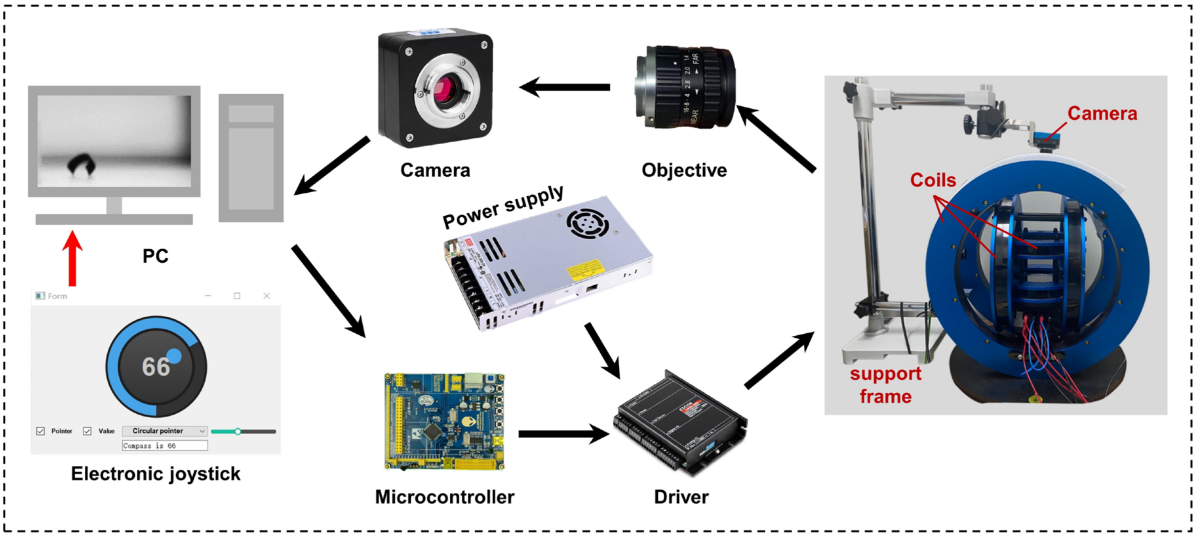

Figure S3. Experimental setup system.

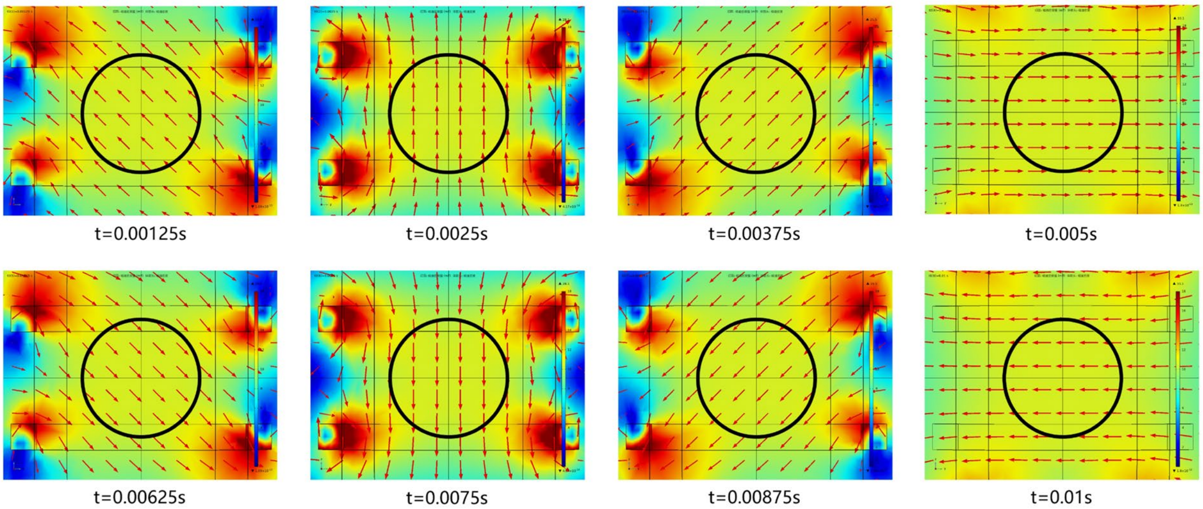

Figure S4. Simulation results of rotating magnetic field.

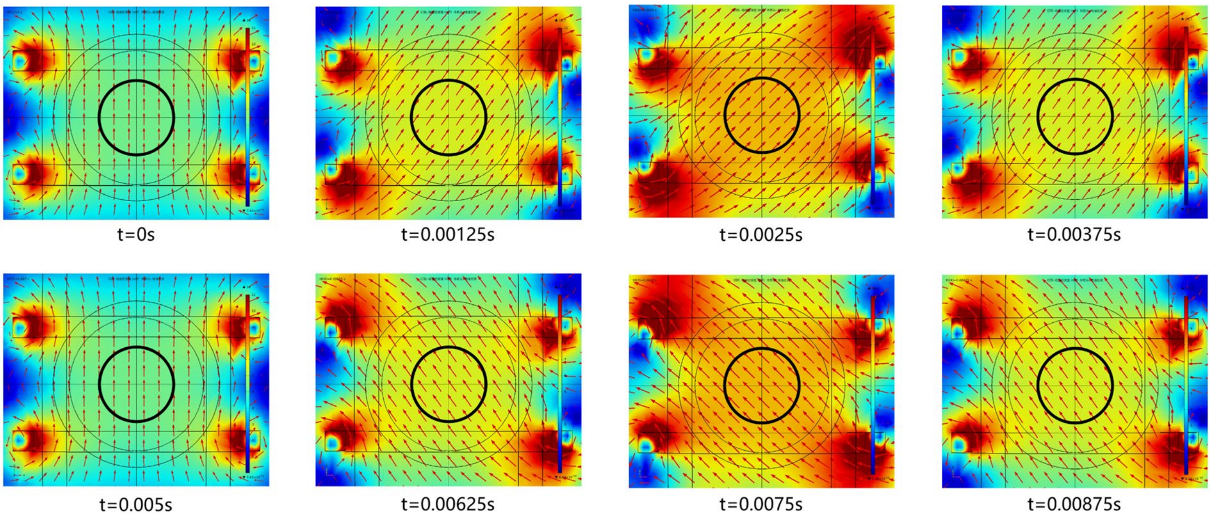

Figure S5. Simulation results of swing magnetic field.

Table S1. Electromagnetic coil parameters

Parameters	X	Y	Z
Number of turns	281	398	262
Equivalent radius (mm)	236	167	110
Wire diameter (mm)	Φ1.95	Φ1.4	Φ1.4
Coil cross section (mm*mm)	35*35	30*30	24*24
Maximum continuous current (A)	10	5	5
Magnetic flux density (mT)	107	107	107
Cold resistance (Ω)	4.8	9.3	4
Inductance (mH)	166	221	58.3

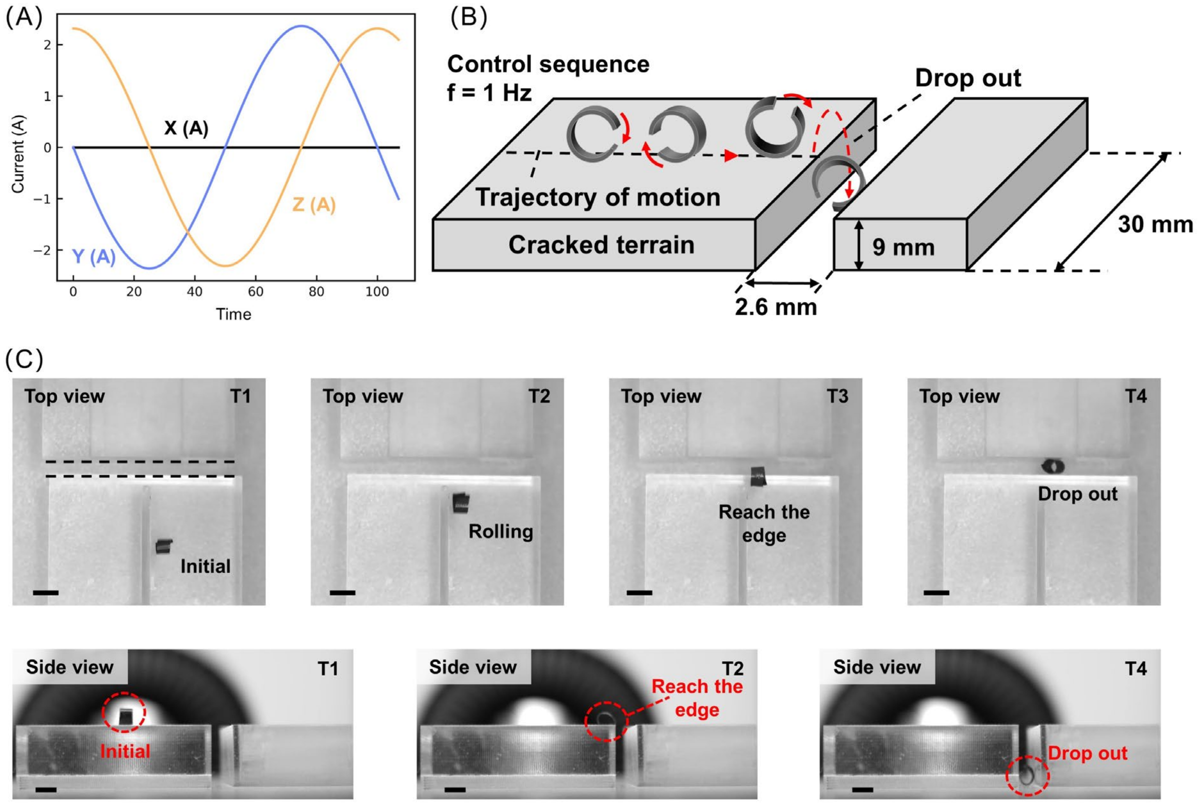

Figure S6. Wheeled millirobots traversing the gap. (A) The currents control signal of generating rotating magnetic field for rolling. (B) Diagram of the millirobot crossing the slit and the schematic of the geometries and sizes in this terrain model. The rolling frequency of the magnetic field is 1 Hz. (C) Millirobot motion state sequence diagram at different time. Scale bar: 5 mm.

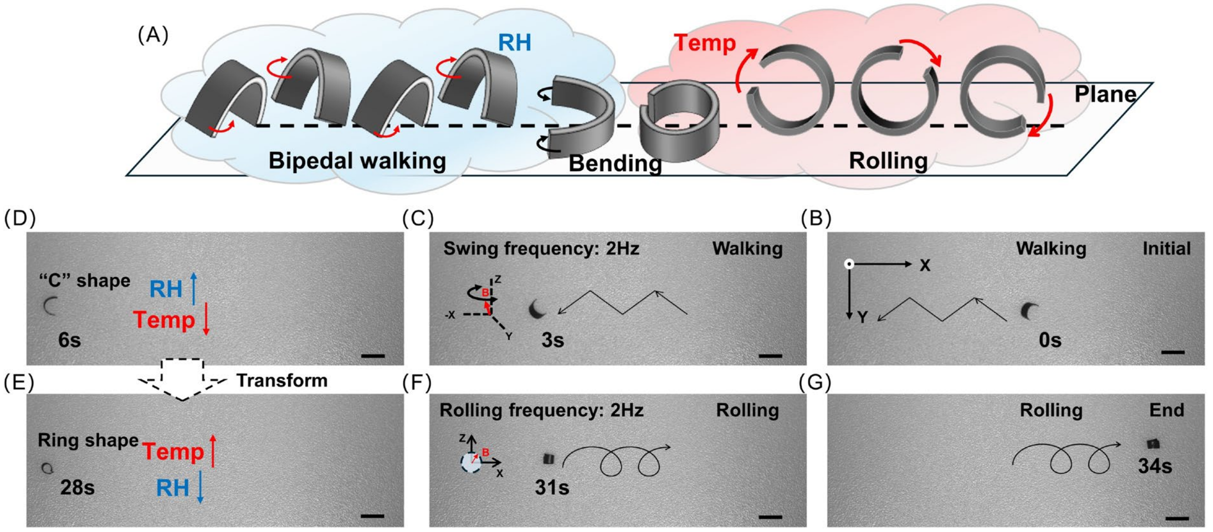

Figure S7. Millirobot dual modality transformation. (A) Schematic representation of the millirobot morphological transformation. (B)-(C) Snapshots of the legged millirobot walking. (D)-(E) Legged millirobot is converted to wheeled millirobot. (F)-(G) Snapshots of the wheeled millirobot rolling. Scale bar: 5 mm.

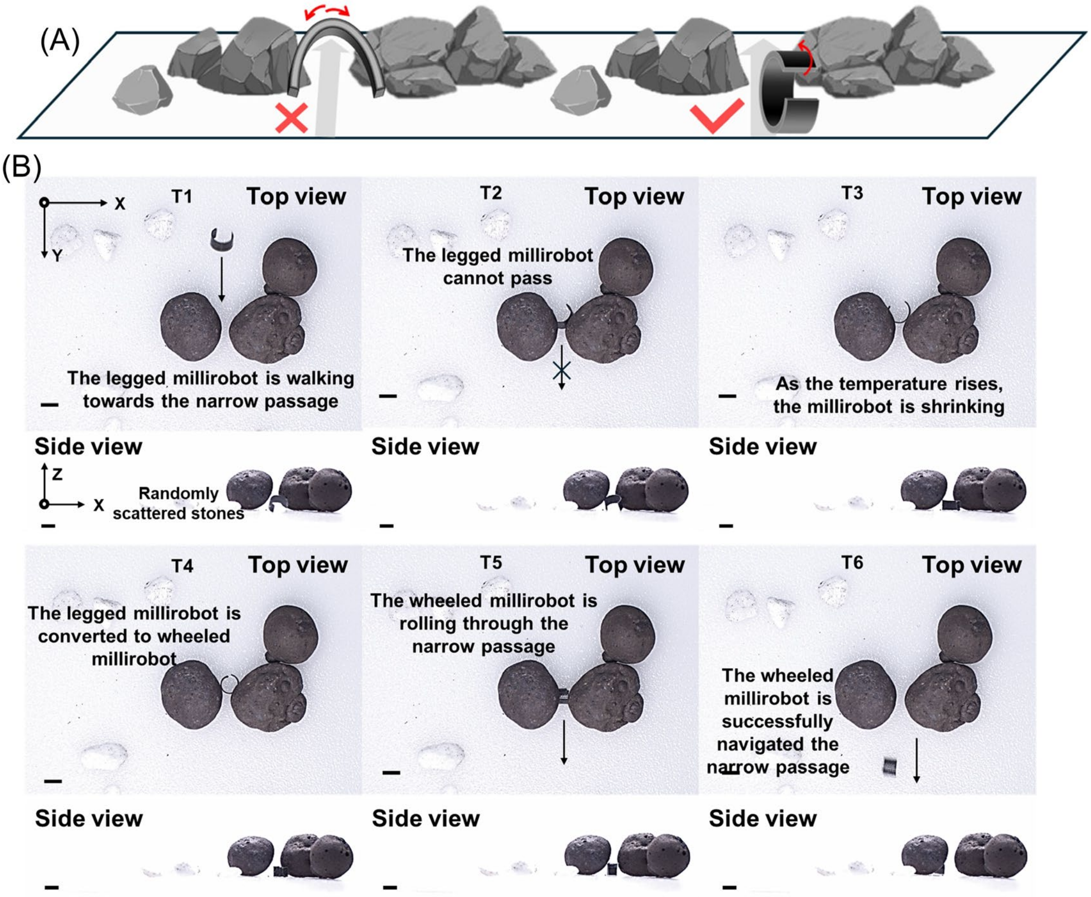

Figure S8. The millirobot passed through unstructured narrow slits based on morphological transformation. (A) Schematic of the mobility of different morphologies of the millirobot. (B) Snapshots of the wheel-legged millirobot passed through unstructured narrow channels. Scale bar: 3 mm. The average radius of the stone is 9 mm.

Table S2. Robot performance comparison table

	Max. Speed	Complex Terrain Adaptability	Controllability	Payload/Function
Wang et al.[1]	Approximately 1.16 Body Lengths/s	Vertical plane, Narrow entries/gaps	Preset signal/manual control	2.5 times its self-weight
Huang et al.[2]	Approximately 1.83 Body Lengths/s	Sand, Muddy, Slope, Gap	Preset signal/manual control	5 times its self-weight
Wang et al.[3]	Approximately 100.6 Body Lengths/s	Jump over the railings of different heights	Preset signal/manual control	Underwater microrobot recovery
Mao et al.,[4]	Approximately 70 Body Lengths/s	Jump over obstacles that are up to 2/3 of your own height	Preset signal/manual control	Cargo transport
Xin et al.[5]	Approximately 0.67 Body Lengths/s	Slit, Stair	Linear closed loop	Drug/cell transport
Our work	Approximately 56 Body Lengths/s	Slope, Curved surface, Stair, Slit, Gap	Nonlinear closed-loop control	13 times its self-weight

References

1. Wang X., Li S., Chang J.C., et al. (2024). Multimodal locomotion ultra-thin soft robots for exploration of narrow spaces. *Nat. Commun.* 15, 6296.
2. Huang H., Feng Y., Yang X. et al. (2022). An insect-inspired terrains-adaptive soft millirobot with multimodal locomotion and transportation capability. *Micromachines* 13, 1578.
3. Mao G., Schiller D., Danninger D., Hailegnaw B., et al. et al. (2022). Ultrafast small-scale soft electromagnetic robots. *Nat. Commun.* 13, 4456.
4. Wang, X., Xia N., Pan C. et al. (2025). Beyond surface tension-dominated water surface jumping. *Nat. Commun.* 16, 3034.
5. Xin Z., Zhong S., Wu A. et al. (2025). Dynamic control of multimodal motion for bistable soft millirobots in complex environments. *IEEE Trans.* Robot. 41, 2662–2676.
